# NeuroSuite for Long‐Term Functional and Structural Studies of Air‐Liquid Interface Cerebral Organoids

**DOI:** 10.1002/advs.202519893

**Published:** 2026-08-06

**Authors:** Belquis Haider, Sagnik Middya, Daniel J. Lloyd‐Davies‐Sánchez, Nino F. Läubli, Sulay Vora, Jakob Träuble, Rohan N. Krajeski, Yuqing Feng, Daniel Duncko, Josiah S. Riley, Rubén Ruiz‐Mateos Serrano, Ole Paulsen, Madeline A. Lancaster, George G. Malliaras, Gabriele S. Kaminski Schierle

**Affiliations:** ^1^ Department of Chemical Engineering and Biotechnology University of Cambridge Cambridge UK; ^2^ MRC Laboratory of Molecular Biology Cambridge UK; ^3^ Institute for Biomedical Innovation, Department of Engineering University of Cambridge Cambridge UK; ^4^ Division of Electrical Engineering, Department of Engineering University of Cambridge Cambridge UK; ^5^ Department of Physiology, Development and Neuroscience University of Cambridge Cambridge United Kingdom

**Keywords:** air–liquid interface cultures, bioelectronics, brain organoids, electrophysiology, microelectrode arrays, multi‐unit analyses, neuronal interfaces

## Abstract

Long‐term electrophysiological monitoring of human cerebral organoids remains challenging, as 3D tissues rapidly develop necrotic cores once diffusion limits are exceeded. Air–liquid interface cerebral organoids (ALI‐COs) overcome this by maintaining thin slices with continuous metabolic exchange, preserving viability, yet no system allows stable, in situ recordings over months. Here, we introduce NeuroSuite, a modular platform for long‐term, spatially resolved neural monitoring of ALI‐COs. Its core, Neuroweb, is an ultrathin, perforated microelectrode array that preserves cellular diversity, evidenced by immunofluorescence and RNA sequencing, while enabling low‐impedance (∼25 kΩ at 1 kHz) recordings. Complementing this, NeuroMaps, our open‐source analysis suite, streamlines signal quality control, spike and field potential analysis, and integration with imaging. NeuroSuite is suitable for capturing maturation‐dependent network dynamics, including potential excitation‐inhibition shifts and oscillatory activity, beyond 180 days. Acute ex vivo rat brain slice recordings further demonstrate its versatility, establishing NeuroSuite as a robust platform for developmental neuroscience, disease modelling, and drug screening.

## Graphical abstract

NeuroSuite provides a modular hardware‐software platform integrating Neuroweb and NeuroMaps to enable long‐term, in situ electrophysiological interrogation of air–liquid interface organoid slices while preserving tissue architecture. Its components can be used together or independently to capture real‐time activity, spatial network dynamics, and maturation over six months, revealing evolving network connectivity and culture‐dependent responses in complex human neural models.

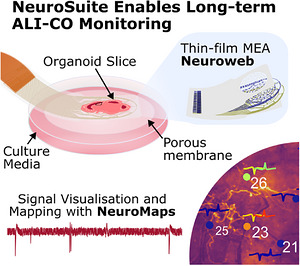

## 1 Introduction

Microelectrode arrays (MEAs) have become indispensable for recording the electrical activity of biological tissues, enabling insights into cellular dynamics and network behavior at high resolution. Originally developed to overcome the invasiveness and low throughput of techniques such as patch clamping in 2D cultures [1], MEAs have evolved in tandem with advances in tissue modelling, most notably, brain organoids.

Derived from human pluripotent stem cells, brain organoids are self‐organizing, three‐dimensional structures that recapitulate key features of human brain morphology and function [2, 3]. They offer unprecedented opportunities to study neurodevelopment [2, 4, 5, 6], neurodegeneration [7, 8, 9], and neurotoxicity [10, 11], while circumventing many of the ethical and translational limitations associated with animal models [12]. However, as organoids grow in size and complexity, oxygen and nutrient diffusion become insufficient to support maturation and viability [13]. Air–liquid interface cerebral organoids (ALI‐COs) adapt organotypic culture methods to overcome diffusion limitations. By exposing the upper surface of organoid slices to air while maintaining medium contact, ALI‐COs enhance nutrient diffusion and gas exchange, supporting neuronal survival and functional axonal outgrowth [14, 15]. Despite these improvements, long‐term in situ electrophysiological monitoring of ALI‐COs remains a significant technical challenge [16, 17]. Conventional MEAs, typically fabricated from rigid planar substrates or emerging 3D [18] and mesh‐like constructs [19], often compromise organoids’ structural integrity, interfere with tissue development, or require re‐plating steps that can damage delicate cytoarchitecture [6, 20, 21, 22, 23]. Furthermore, the mechanical mismatch between stiff electrodes and soft brain tissue can disrupt natural neuronal organization or alter signaling, further confounding results [24, 25, 26].

Flexible and stretchable MEAs, designed with form factors that better accommodate tissue mechanics and fabricated from softer, more compliant materials than glass [27, 28, 29, 30], including SU‐8, parylene‐C, styrene‐ethylene‐butylene‐styrene (SEBS), and polydimethylsiloxane (PDMS), have improved interfacing in cardiac cells and neural tissue in vivo. However, their adaptation for brain organoid research is still emerging. Recent approaches, such as introducing mesh nanoelectronics into brain organoids’ morphogenesis, enable chronic single‐cell recordings from within the tissue, but are incompatible with slicing protocols due to the risk of device damage [30]. Other approaches, including kirigami‐inspired [31] or shell‐type arrays [29], improve experimental flexibility and adaptability, but often involve complex assembly and handling. Moreover, their electrode spans of ∼1 mm are poorly suited for ALI‐COs, which typically grow up to ∼5 mm and can survive more than one year [32, 33, 34].

To address these limitations, we developed NeuroSuite, a modular platform for the long‐term, non‐invasive electrophysiological monitoring of ALI‐COs and organotypic brain cultures. NeuroSuite combines Neuroweb, a flexible, ultrathin (4 µm), perforated microelectrode array, with NeuroMaps, an open‐access software suite for signal quality assessment, spatial localization, and longitudinal analysis of spikes and local field potential (LFP). Neuroweb's perforated design features a low‐fill ratio (30%), enabling excellent conformability and mechanical compliance while preserving nutrient and gas exchange. The radial distribution of the 32 electrodes is ideal for capturing neural activity as the 300 µm thick slices mature from 500 µm to 5 mm in diameter, and the material stack of gold and the conducting polymer poly(3,4‐ethylenedioxythiophene):polystyrene sulfonate (PEDOT:PSS) enables high signal‐to‐noise ratio electrophysiology (∼16 dB) from localized electrode sites (∼30 µm in diameter).

We validate Neuroweb through comprehensive functional characterization and long‐term electrophysiological recordings of human ALI‐COs and acute rat brain slices. Using NeuroMaps, we tracked and spatially mapped impedance, spike rates, synchrony, bursting patterns, and waveform features (e.g., full width at half maximum, amplitude, and rate of depolarization), observing stable impedance for up to six months, while waveform features changed dynamically, reflecting emerging neural connectivity. Crucially, NeuroMaps revealed refinement in spike population dynamics, which is consistent with neuronal maturation and increased inhibitory regulation. Additionally, we observed changes in the aperiodic exponent of the local field potential (LFP) power spectra, an indicator of refinement in the network excitation‐inhibition balance [32], along with increased burst regularity and spatially localized connectivity.

NeuroSuite establishes a powerful, integrated framework for long‐term functional studies of human brain organoids, revealing a level of insight that would be inaccessible using conventional, short‐term devices, and thus provides an ideal platform for disease modelling, developmental neuroscience, and high‐throughput drug screening.

## 2 Results

### 2.1 NeuroSuite Offers an Accessible and Scalable Framework for Electrophysiological Investigations

**FIGURE 1:**
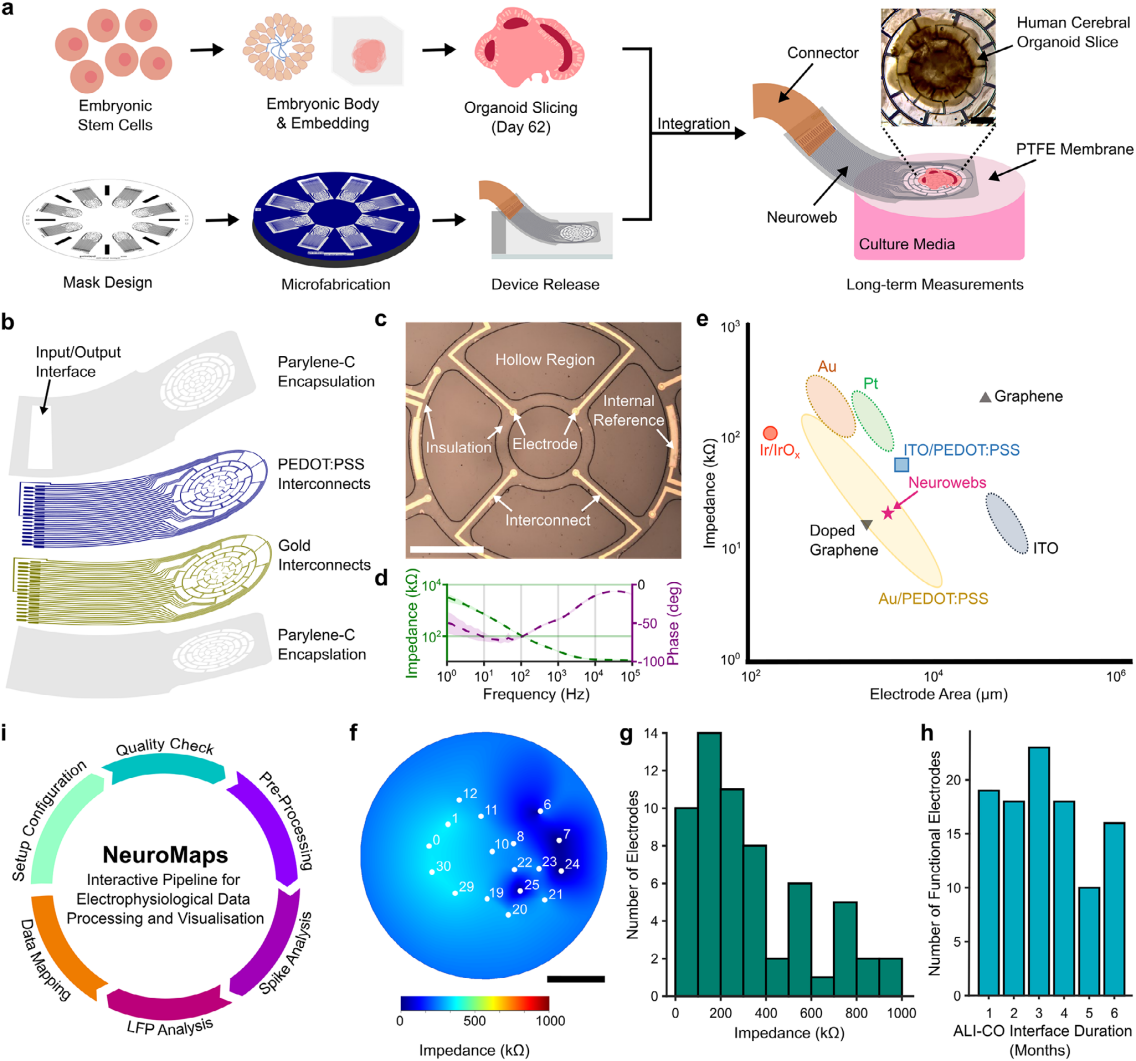
The NeuroSuite pipeline: device design, fabrication, and functional characterization for long‐term integration with air–liquid interface cerebral organoids (ALI‐COs). (a) Overview of the modular workflow, showing generation of cerebral organoid slices from embryonic stem cells, scalable microfabrication of Neuroweb devices, and integration for long‐term culture at the air–liquid interface. Inset presents a brightfield image of the ALI‐CO just after slicing and interfacing (days in vitro; DIV 62). (b) Schematic of Neuroweb's multi‐layer structure: 2 µm thin top and bottom parylene‐C encapsulation layers, 600 nm PEDOT:PSS electrode layer, and 100 nm gold interconnect layer. (c) Optical micrograph highlighting Neuroweb's perforated, ultrathin architecture, enabling efficient nutrient exchange and conformal interfacing with ALI‐COs. SEM images are provided in Figure S4. (d) Bode plot depicting impedance and phase profiles of PEDOT:PSS‐coated gold electrodes (1 Hz–100 kHz), showing capacitive behavior at low frequencies and resistive behavior at higher frequencies. (e) Benchmark comparison of Neuroweb's electrode impedance at 1 kHz in PBS with previously reported materials, including Au [35, 36, 37], Au/Pt [31, 36], Pt [38, 39], Ir/IrOx [40], Si [40], ITO [41, 42], and ITO/PEDOT:PSS [43]. (f) Illustration of NeuroMaps functions, enabling analysis of Neuroweb data including signal filtering, quality tracking, spike detection, spatial mapping, and electrophysiological feature extraction. (g) Impedance heatmap of all electrodes mapped onto the array layout using NeuroMaps. (h) Histogram of impedance values from two devices integrated with organoids, demonstrating a Gamma distribution with most electrodes remaining below 200 kΩ at 1 kHz. (i) Histogram quantifying the number of functional electrodes across six months of continuous organoid interfacing, assessed via impedance, standard deviation, median absolute deviation, and power spectral density (see Materials and Methods). Scale bars: (a) 1 mm, (c) 500 µm, (g) 1 mm.

NeuroSuite is designed for non‐invasive, long‐term electrophysiological monitoring of air–liquid interface cerebral organoids (ALI‐COs), addressing the critical need for stable functional readouts without compromising tissue viability. Our modular workflow (Figure 1a) integrates cerebral organoids or ex vivo slices with Neuroweb, a flexible organic microelectrode array fabricated using scalable microfabrication techniques (Figures S1 and S2).

Neuroweb is mounted on standard 0.4 µm pore diameter polytetrafluoroethylene (PTFE) cell culture inserts previously validated for long‐term ALI‐CO culture [14], and housed within a customizable, autoclavable polycarbonate adapter and enclosure. This configuration supports stable air–liquid interface culture and routine media exchange while minimizing evaporation and contamination (Figure S3). Biocompatible sealants and the detachable printed circuit board (PCB) adapter allow incubator‐compatible in situ recordings without compromising electronics.

Each array comprises 32 electrodes (∼30 µm in diameter) and two internal references, encapsulated between two ultrathin parylene‐C layers (2 µm in thickness each) (Figure 1b,c; Figure S4). The electrodes are coated with PEDOT:PSS [36], a mixed ionic‐electronic conductor that increases the capacitance at the electrode/electrolyte interface [33], yielding a low electrode impedance of 25 ± 9 kΩ at 1 kHz in phosphate‐buffered saline (PBS) at 25°C (Figure 1d,e).

Electrodes are arranged in five concentric rings, with a radial spacing of 600 µm, optimized for both multi‐unit activity (MUA) and local field potential (LFP) recordings. The 4.4 mm electrode span accommodates organoids growing from a diameter of ∼700 µm on the day of slicing and integration (DIV 62) up to a diameter of ∼5 mm at later stages. Approximately 70% of the central area is perforated to ensure unobstructed nutrient diffusion and reduce the risk of necrosis or cellular stress at the device‐tissue interface. The resulting bending stiffness (∼0.72 pNm2, estimated using classical beam theory; Text S1) is sufficiently low to minimize the mechanical mismatch with the 300 µm thick organoid slices [26], while remaining robust enough for manual device handling and interfacing.

Complementing Neuroweb, we developed NeuroMaps, a user‐friendly MATLAB‐based platform that enables electrode and signal quality assessment using established quality metrics [34], with integrated tools for longitudinal spike and LFP analyses and spatial mapping (Figure 1f). Our spatial mapping of impedance values across Neuroweb (Figure 1g) revealed a gamma‐like distribution, with most electrodes exhibiting an impedance of under 200 kΩ at 1kHz (Figure 1h). Notably, over 50% of the electrodes remain functional after six months of continuous interface and multiple ethanol device clean‐up cycles (Figure 1i).

### 2.2 Neuroweb Supports Long‐Term Growth and Maturation of ALI‐COs

To assess the biocompatibility and suitability of Neuroweb for long‐term organoid interfacing, we cultured ALI‐COs directly on the device. Cortical organoids were generated and embedded in Matrigel following established protocols [14], then sliced on DIV 59 to 62 to preserve structural integrity prior to transfer onto the Neuroweb platform. Cultures were maintained at the air–liquid interface for an additional 48 days (up to DIV 110), as detailed in the Materials and Methods section, after which samples were fixed and processed for imaging.

**FIGURE 2:**
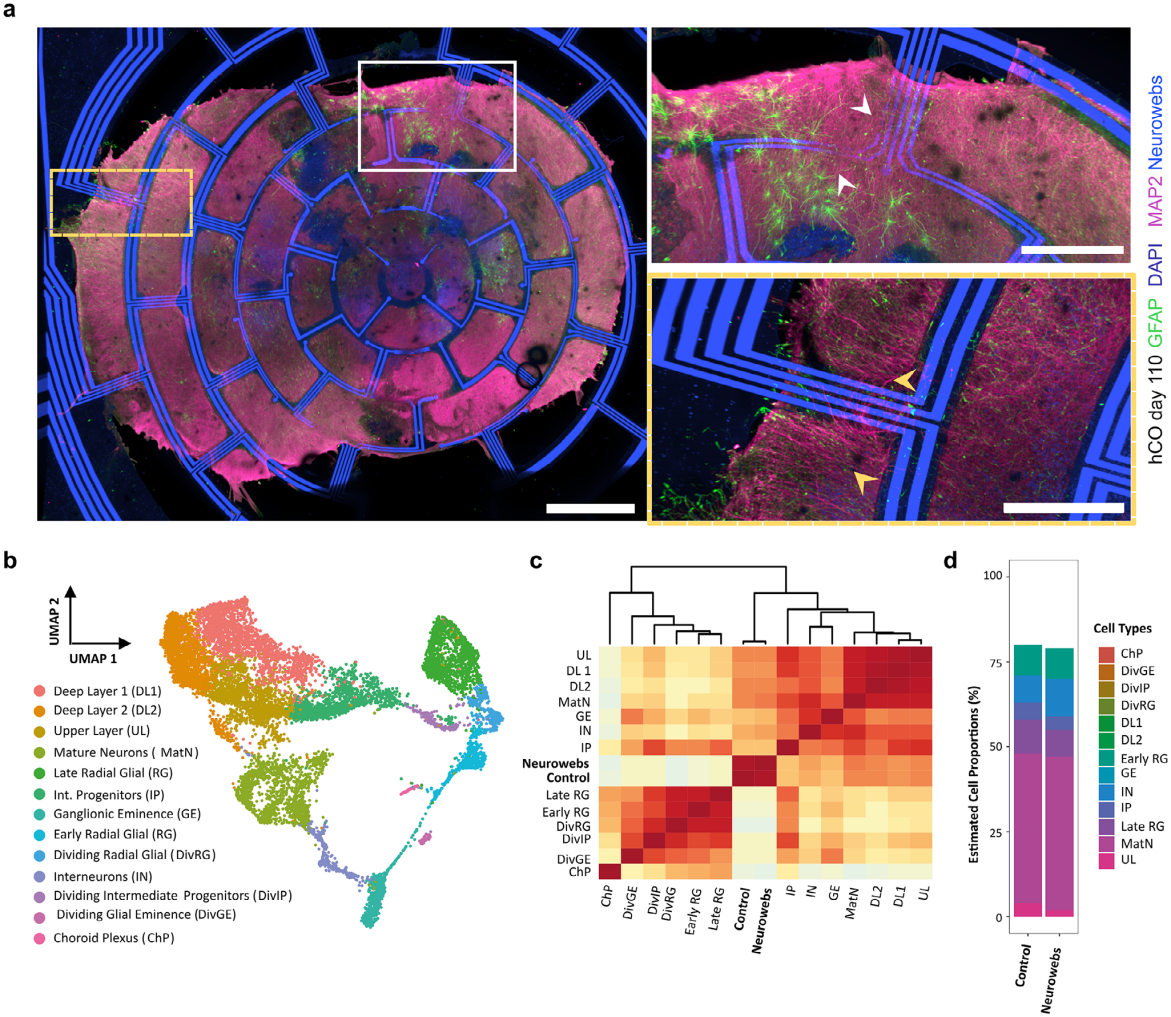
Neuroweb supports robust growth, structural organization, and cellular diversity in ALI‐COs. (a) Confocal immunofluorescence image of a human cerebral organoid cultured on Neuroweb for 48 days (DIV 110). Nuclei are stained with DAPI (dark blue), neurons with MAP2 (magenta), and glial cells with GFAP (green); Neuroweb's conductive tracts appear in bright blue due to laser light scattering from the gold layer. The top‐right inset highlights organoid encapsulation of the device (white arrows), while the bottom‐right inset shows radially aligned MAP2‐positive dendrites (yellow arrows), indicating directional neuronal outgrowth. (b) Uniform Manifold Approximation and Projection (UMAP) plot of single‐cell RNA sequencing data from Neuroweb‐supported organoids on DIV 110, identifying 13 major cell clusters. Full cluster annotations are shown in Figure S7. (c) Hierarchical clustering dendrogram reveals dominant mature neuronal populations: upper‐layer (UL), deep‐layer 1 and 2 (DL1, DL2), mature neurons (MatN), ganglionic eminence (GE), interneurons (IN), and intermediate progenitors (IP), with only minor representation of progenitor and non‐neuronal types, including late, early, and dividing radial glia (LateRG, EarlyRG, DivRG), dividing IPs (DivIP), dividing GE cells (DivGE), and choroid plexus (ChP). (d) Bar plot showing cell‐type proportions from deconvolved scRNA‐seq data for Neuroweb‐grown organoids and controls (cultured on standard cell‐culture inserts), demonstrating comparable cellular composition. (c,d) n = 2 biological replicates per condition. Scale bars: (a) main panel: 1 mm; insets: 400 µm.

Immunofluorescence combined with DAPI nuclear staining confirmed robust organoid expansion across the array, indicating preserved cellular architecture and no overt morphological signs of cell death (Figure 2a). This was further confirmed through live/dead staining, where no significant difference was detected between samples grown on Neurowebs and corresponding controls (Figure S5). The perforated design and minimal insulating footprint of Neuroweb permitted tissue integration, supporting dense networks of neurons (MAP2) and astrocytes (GFAP) that enveloped the electrode architecture (Figure 2a, top inset), while MAP2 staining revealed radially aligned dendrites indicative of healthy, directional neuronal outgrowth (Figure 2a, bottom inset), Further immunofluorescence imaging confirmed the presence of axonal projections (SMI312) extending throughout the slice (Figure S6), with sparse unlabeled regions across the micrographs likely corresponding to non‐neuronal cell types. These observations reflect the expected heterogeneity of minimally guided organoid cultures, mirroring in vivo variability, with isolated darker areas likely constituting common imaging artifacts experienced when mounting large organoid samples which are attached to flexible bioelectronic devices, tissue folds, or limitations in dye penetration.

To further assess the cellular composition of Neuroweb‐supported organoids and complementary to the immunofluorescence analyses, we performed bulk RNA sequencing and compared the results to single‐cell data from standard ALI‐COs. Uniform Manifold Approximation and Projection (UMAP) analysis identified thirteen major cell types, including upper and deep‐layer excitatory neurons (UL, DL1, DL2), interneurons (IN), radial glia (RG), intermediate progenitors (IP), and choroid plexus cells (ChP) (Figure 2b). Crucially, despite the inherent biological heterogeneity of cerebral organoids, our results indicate that Neuroweb‐grown ALI‐COs preserved cellular diversity comparable to controls cultured on standard inserts (Figure 2c,d) and featured similarly mature cell‐type profiles to pre‐existing datasets (Figure 2c; Figure S7), thus confirming that the device did not perturb natural developmental trajectories.

Finally, to assess the impact of Neuroweb's perforated architecture, we compared it to a conventional micro‐electrocorticographic array (µECoG) previously validated in vivo [44]. After 69 days of culture, ALI‐COs placed on µECoG devices started to disintegrate. Corresponding immunostaining revealed a stark contrast in biocompatibility. While Neuroweb, with a 30% fill ratio, supported extensive neuronal (MAP2) and axonal (SMI312) networks and healthy glial morphology, organoids cultured on the less perforated µECoG (70% fill ratio) exhibited minimal growth and displayed large dark regions indicative of impaired nutrient diffusion and structural disruptions (Figure S8).

### 2.3 NeuroMaps Enables the Visualization of Network Dynamics from ALI‐COs and Acute Brain Slices

Following validation of Neuroweb's suitability for long‐term culturing of ALI‐COs, we used NeuroMaps for longitudinal analysis and visualization of spontaneous and evoked electrophysiological activity. Pre‐processing included impedance‐based electrode selection (<1 MΩ), signal quality assessment, and powerline noise filtering (detailed in Materials and Methods and Figure S9).

**FIGURE 3:**
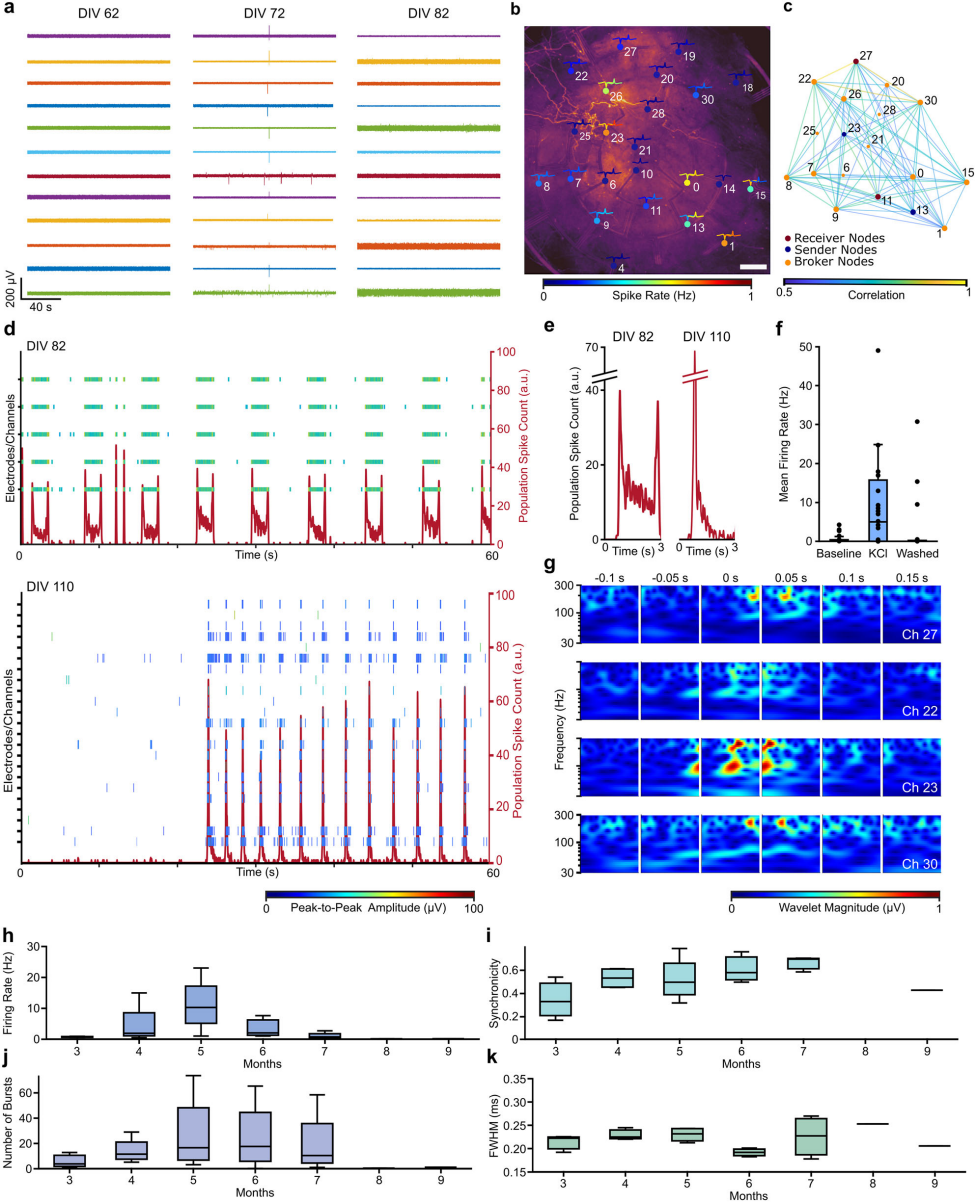
Longitudinal tracking of electrophysiological maturation in ALI‐COs. (a) Representative raw traces of spontaneous activity recorded from an organoid slice at DIV 62, 72, and 82, illustrating progressive increases in network‐level spiking activity. (b) Detected action potentials overlaid on a corresponding immunofluorescence image of the ALI‐CO (DIV 110) using NeuroMaps, enabling spatial co‐registration of electrical and anatomical data. The slice culture is labelled sparsely with GFP using a Sendai virus. (c) Functional connectivity map based on STTC analysis, identifying key network nodes; electrode 27 is classified as a receiver and electrode 22 as a broker. (d) Raster plots and corresponding population spike rate plots at DIV 82 and DIV 110 reveal increasing temporal precision and network synchrony during maturation. The color scale indicates spike peak‐to‐peak voltage. (e) Zoomed‐in traces of population‐wide spiking events highlighting sharper and more transient responses at DIV 110 compared to DIV 82. (f) Validation of neuronal activity via chemical stimulation with 50 mM KCl, which significantly increased spike activity across 11 channels (n  =  2 organoids). (g) Continuous wavelet transform analysis revealing broadband gamma bursts (30–300 Hz) during population activity. Temporal alignment of bursts between channels 27 and 22, and channels 30 and 23, is consistent with their inferred roles in (c). (h–k) Longitudinal quantification of key electrophysiological metrics: (h) mean firing rate peaks at ∼month 5 before declining; (i) network synchrony increases progressively through month 7; (j) burst frequency follows a similar pattern to the firing rate; (k) FWHM remains relatively stable. Scale bar: (b) 500 µm.

Representative raw broadband signals are shown across developmental timepoints (Figure 3a; Figure S10). To quantify coordinated neuronal activity, both positive and negative polarity spikes were extracted from band‐pass filtered signals (300–3000 Hz) using a threshold of 4.5× the noise standard deviation of the signal [45], a 2‐ms refractory period, and a 1 ms full width at half maximum (FWHM) criterion to exclude artefacts. Phase plots of depolarization characteristics are shown in Figure S11.

Early recordings (DIV 62) revealed sparse, uncoordinated neuronal spiking activity that transitioned to broad network‐wide activity by DIV 82. At DIV 72, near‐synchronous spikes across multiple electrodes suggest an increase in gap junction‐mediated synchrony. Meanwhile, slight temporal delays between signals on spatially separated electrodes indicate activity from distinct neuronal populations, validating Neuroweb's capacity to resolve inter‐population dynamics. NeuroMaps localized spike origins (DIV 110) at the electrode level and co‐registered waveforms with the uploaded immunofluorescence image (Figure 3b). Given the high cellular density of the tissue, these spikes reflect multi‐unit or multi‐source activity within densely packed networks.

To assess functional connectivity [46], we quantified connection strength by computing spike‐time tiling coefficients (STTC) [47] between all electrode pairs (nodes). Directionality was inferred based on spike timing relationships as described previously [48] and as outlined in Materials and Methods. In brief, a connection from node i to node j was assigned when spikes in i consistently preceded those in j. For each node, we quantified outgoing (k_out_) and incoming (k_in_) connections as the weighted sum of significant directed spike propagation. Nodes were classified as senders (k_out_/k_in_> 0.8), receivers (k_in_/k_out_>0.8), or brokers (otherwise) (Figure 3c) [14, 48]. STTC strength peaked at an interelectrode distance of ∼1360 µm, with moderate correlations at ∼1740 µm and weaker correlations around ∼1640 µm, consistent with Neuroweb's electrodes’ spatial distribution and suggesting spatially structured, long‐range coordination in activity (Figure S12). Raster plots and population traces, displayed in Figure S13 and derived as detailed in Materials and Methods, captured a transition from prolonged and reverberant activity (DIV 82) to transient, well‐defined events with sharply decaying profiles (DIV 110) (Figure 3d,e). The observed shift may reflect early maturation of inhibitory circuitry, in line with previous observations linking it to an increased contribution of GABAergic interneurons during cortical maturation [49, 50, 51]. In our study, the signal origin was confirmed by depolarizing two organoids with potassium chloride (KCl) [52], which significantly increased activity before returning to baseline upon washout (Figure 3f). Furthermore, to verify the capabilities of NeuroMaps to extract biological features, we performed acute pharmacological measurements with Tetrodotoxin (TTX) and bicuculline. As expected, TTX treatment led to a significant decrease in spike rate and amplitude recorded from DIV 140 organoids, while the DMSO control did not affect organoid behavior (Figure S14). Furthermore, while acute treatments with bicuculline compared to the DMSO control demonstrated weak yet consistent trends in terms of spike rate, amplitude, and synchrony (Figure S15), these were non‐significant. This may be because the GABAergic switch had not yet occurred, or because the bicuculline dose was too low to elicit a significant response.

We additionally analyzed low‐frequency oscillatory dynamics using a continuous wavelet transform (CWT) to capture both the temporal and spectral characteristics of population activity (Figure 3g). This revealed co‐occurring bursts in the broadband gamma range (30–300 Hz) during episodes of high‐frequency neuronal spiking activity. These oscillations, typically associated with local circuitry spanning 250–500 µm in vivo [53, 54], are potentially driven by excitatory and inhibitory interactions shaping population‐level output in ALI‐COs. Notably, the temporal lag between these oscillations on channels 22, 23, 27, and 30 aligns with their inferred functional roles from the STTC map, where channel 23 is identified as a sender, channel 27 as a receiver, and the others as brokers (Figure 3c). These findings were independently replicated (Figure S16), where further spike feature analysis depicted a change in the peak‐to‐peak amplitude of the spikes during propagation, whereas the full width at half maximum remained relatively constant.

Beyond the snapshot analyses presented above, we analyzed 5‐min‐long recordings acquired from eleven cerebral organoids across partially overlapping time windows. The average spike rates increased from 0.9 ± 0.2 Hz at month 3 post‐seeding to a peak of 11.2 ± 9 Hz at month 5, followed by a progressive decline to 3.6 ± 1 Hz in month 6 and 1.3 ± 1 Hz in month 7 (Figure 3h). Beyond this point, activity fell below 0.3 Hz, correlating with a significant drop in neuronal activity (<10 electrodes detected activity). Relatedly, network synchronicity rose from 0.3 ± 0.2 to 0.5 ± 0.2 between months 3 and 5 (Figure 3i), while burst frequency increased from 5.8 ± 6.1 to 27.4 ± 31.8 bursts per 5‐min interval (Figure 3j), both indicative of maturing connectivity and coordinated neuronal spiking activity. Interestingly, despite these functional changes, the FWHM values remained relatively stable across all time points (Figure 3k), suggesting that the biophysical properties of the spike waveforms were maintained throughout extended culture. This consistency underscores Neuroweb's ability to provide a minimally invasive, stable interface for chronic recordings, preserving signal fidelity even as network‐level activity evolves.

To further demonstrate Neuroweb's versatility, we performed acute rat brain slice ex vivo recordings capturing the transition from sparse spiking activity at DIV 10 to highly synchronized activity on DIV 20 (Figure S17). The electrophysiological trajectories observed across both acute and long‐term ALI‐CO recordings suggest age‐dependent modulation arising from intrinsic maturation, network remodeling, and/or evolving tissue‐device interface properties. These findings thus underscore the importance of longitudinal electrophysiological monitoring for resolving non‐linear developmental trajectories that may be obscured by snapshot analyses, positioning Neuroweb as a robust platform for studying dynamic network evolution in complex in vitro neural systems.

### 2.4 NeuroSuite Reveals Age‐Dependent Shifts in Low‐Frequency Network Dynamics

To extend our electrophysiological characterization of human‐derived ALI‐COs, we expanded the analysis to the low‐frequency components of recorded signals, offering complementary insight into large‐scale network behavior. While high‐frequency activity (300–3000 Hz) captures spatially localized action potentials, low‐frequency LFPs (0.5–300 Hz) reflect ensemble‐level neuronal dynamics across broader spatial domains, providing valuable markers of network synchronization, oscillatory coordination, and temporal structure.

**FIGURE 4:**
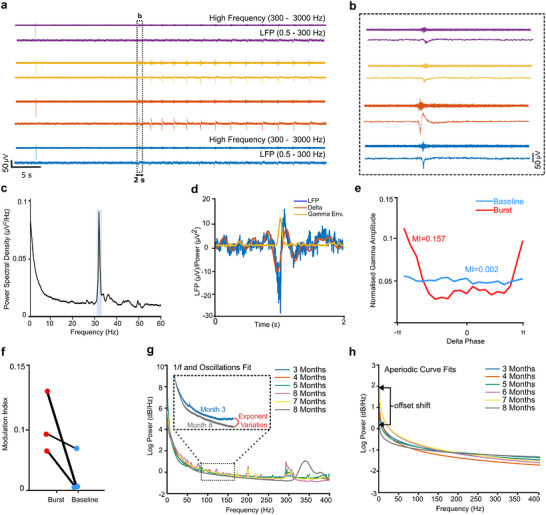
Spectral and temporal features of neural activity reveal coordinated network dynamics and maturation in ALI‐COs. (a) Representative traces recorded at DIV 110 from multiple electrodes (color‐coded) showing broadband high‐frequency activity (300–3000 Hz, top traces) and low‐frequency LFPs (0.5–300 Hz, bottom traces). (b) Zoomed‐in view of a network burst reveals temporal alignment between spiking and LFP oscillations. (c) Power spectral density (PSD) analysis of a mature organoid shows a peak in gamma activity centered at 35–40 Hz. (d) Decomposition of the LFP into broadband gamma amplitude (yellow) and delta phase (orange) illustrates cross‐frequency modulation. (e) PAC analysis reveals elevated modulation index (MI = 0.157, black) during network bursts compared to baseline (MI = 0.002, red). (f) Average PAC values across multiple organoids confirm that gamma–delta coupling is enhanced during synchronized network events. (g,h) Parameterized PSD fits show age‐dependent reductions in the aperiodic offset and exponent, consistent with neural network maturation. The inset in (g) depicts the curves’ aperiodic (1/f) exponential variation over maturation.

Focusing on mature ALI‐COs at DIV 110, we decomposed broadband recordings into LFP components and compared them to their corresponding high‐frequency traces (Figure 4a). A magnified view of a population burst (Figure 4b) revealed a precise temporal alignment between high‐frequency spiking and low‐frequency oscillations, indicating coordinated activity across multiple spatial and temporal scales.

To quantify these dynamics, we performed power spectral density (PSD) analysis of the LFP signals (Figure 4c). A distinct spectral peak emerged in the gamma range (30–60 Hz), which has previously been linked to synchronized population spiking activity [55]. Gamma power was notably elevated during spiking bursts, reinforcing its value as a surrogate marker for coordinated ensemble activity.

Given the known interaction between fast (high‐frequency gamma, 100–400 Hz) and slow (delta, 1–4 Hz) oscillations in cortical circuits, we next examined phase‐amplitude coupling (PAC) to determine whether high‐frequency gamma activity was modulated by slower delta rhythms [6, 56, 57, 58]. Signals were filtered using a finite impulse response (FIR) filter to extract the gamma amplitude and delta phase (Figure 4d). The modulation index (MI), a commonly used metric for quantifying PAC, was significantly elevated during synchronized network events compared to baseline periods (MI: 0.157 vs. 0.002, Figure 4e,f). This suggests that fast dynamics are temporally structured by slow‐wave activity, which is a hallmark of hierarchical organization and functional maturation in both in vivo and in vitro neural systems [59, 60].

We further tracked changes in aperiodic and periodic spectral features using spectral parameterization algorithms [32] embedded in NeuroMaps. In cortical ALI‐COs, both the aperiodic exponent and offset increased up to month 7, suggesting a progressive development of ALI‐CO inhibitory circuits and enhanced population‐level connectivity. These trends may reflect the maturation of GABAergic signaling or refinement in the excitatory‐inhibitory balance, however, given continuing uncertainties at earlier stages (DIV 140, Figure S15), additional long‐term investigations into these changes are required to confirm their nature (Figure 4g,h) [32].

Lastly, we applied this approach to track oscillatory dynamics over 24‐h recordings, observing not only a maturation‐associated increase in gamma power (Figure S18) but also fluctuations in spectral power at ∼14 h of recording (Figure S19). This may indicate intrinsic metabolic cycling within organoids, highlighting their potential as in vitro models for long‐term neural metabolic and rhythm research.

## 3 Discussion

In this study, we developed NeuroSuite, a next‐generation platform for stable, high‐resolution electrophysiological recordings from ALI‐COs over six months. At its heart is Neuroweb, a flexible, perforated organic microelectrode array that supports the dynamic growth of sliced cerebral organoids (up to 5 mm in diameter) while preserving metabolic exchange and tissue integrity. Paired with NeuroMaps, a user‐friendly MATLAB‐based toolbox, the system enables interactive assessment, visualization, and quantification of evolving neural dynamics across multiple frequency bands.

While traditional rigid and penetrating microelectrode arrays provide valuable functional insights into organoids [6, 14] through their high‐throughput capabilities [48, 61] and improved signal‐to‐noise ratios [62], they often fall short in chronic use due to their rigidity and invasiveness, which can compromise tissue architecture [24, 25]. Neuroweb overcomes these limitations with an ultrathin, low‐fill design that supports oxygen and nutrient diffusion without compromising spatial resolution. Unlike flexible mesh arrays based on SU‐8 [30, 31], which are accompanied by cytotoxicity concerns [63, 64], the parylene‐C substrate supports electrode encapsulation and spontaneous organoid growth at the air–liquid interface. Immunofluorescence and RNA‐sequencing confirmed biocompatibility, enabling a stable, long‐term interface for high‐fidelity signal acquisition. The non‐invasive nature of our design was further verified through live/dead staining, where no significant differences between ALI‐COs cultured on Neurowebs and slices maintained on standard membranes were observed. However, due to the inherent heterogeneities of unguided organoid cultures and corresponding batch‐to‐batch variability, effects caused by potential differences in baseline tissue quality cannot be fully excluded.

Next, we confirmed the biological origin of the quantified electrophysiological recordings using a series of functional and pharmacological treatments. KCl stimulation expectedly led to a clear increase in spiking activity, with the signal depleting again following repeated washouts. Similarly, acute TTX treatments induced statistically significant differences in both spike rate and amplitude, while such changes were not observed for DMSO‐controls. However, the observed temporal lag in the biological response and the limited recovery suggest that increased doses or, due to the high density of the tissue, longer incubations and washing cycles may be needed to fully block and reinstate the voltage‐gated sodium channels. Finally, acute bicuculline treatment of 140 DIV organoids included consistent yet not significant changes in spike rate, amplitude, and synchrony, thus suggesting that a GABAergic switch had not yet occurred at this stage or that the applied bicuculline dose may have been too small to elicit a significant response. This is also complicated by the inherent heterogeneity of unguided cerebral organoids highlighted above, where the achievement of developmental milestones varies between samples and where biological responses may be affected by broader tissue compositions. Accordingly, future work may aim to systematically investigate the impact of such heterogeneities, including variations in glial cell distribution or the occurrence of local apoptosis and the spatiotemporal remodeling of samples, on local electrophysiological properties.

Our experimental setup is further optimized through a modular, autoclavable 3D‐printed holder that seamlessly integrates with standard cell‐culture components and imaging systems. This design enables in situ recordings while reducing handling complexity [28, 31]. It also eliminates the need for manual gluing steps for device stabilization and assembly [65]. PEDOT:PSS‐coated gold electrodes further enhance Neuroweb's performance, achieving superior signal‐to‐noise ratios relative to platinum [30, 38, 39], ITO [41, 42], uncoated gold [31, 35, 36], and graphene [38, 66] while maintaining small electrode diameters (30 µm) suitable for mesoscale recordings across dense neural populations. Furthermore, the complete setup incorporating the holder and the flexible electronics can be produced at comparable costs to commercial alternatives, while its streamlined fabrication procedure and compatibility with upscaling may enable further cost reductions.

To our knowledge, NeuroSuite is the first platform to integrate minimally invasive, chronic neural interfacing with a user‐friendly analytical suite, unlocking scalable, high‐throughput interrogation of organoid network dynamics over time. NeuroMaps provides a graphical interface integrating validated standards [6, 37, 47, 67, 68, 69], including tools for signal quality assessment, spike and burst detection, network mapping, and low‐frequency aperiodic, periodic, and oscillatory analyses, accessible with no prior coding experience. Over six months of stable interface, NeuroSuite enabled robust tracking of network activity in maturing ALI‐COs, capturing dynamic changes in spike frequency, bursting patterns, and synchronicity. The stable, minimally invasive interface makes Neuroweb well‐suited for future studies elucidating the mechanisms underlying these age‐dependent changes and shifts in low‐frequency oscillatory behavior.

Future extensions could integrate larger stimulation electrodes [70, 71], biosensors [27], or micro‐light‐emitting diodes (µ‐LEDs) [72] for multimodal interfacing and neuromodulation, with investigations elucidating long‐term electrode‐tissue interactions also offering novel insights relevant for bioelectronic material development. On the analytical side, embedding scalable data handling frameworks and machine learning‐based spike sorting into NeuroMaps would enable high‐throughput applications across increasingly complex organoid models. Additionally, implementing PEDOT:PSS‐only electrode regions would further enable artifact‐free imaging in the z‐direction, supporting volumetric evaluation of the ALI‐COs as they encapsulate Neuroweb. Together, these advances position NeuroSuite as a transformative platform for long‐term, in situ electrophysiology in ALI‐COs. With its modular design and seamless integration of hardware and analysis, NeuroSuite opens new avenues for decoding circuit‐level development and dysfunction in neurodevelopment, ageing, neurodegeneration, thereby contributing to the deployment of more predictive drug screening in human‐relevant models.

## 4 Materials and Methods

### 4.1 Flexible Device Design and Fabrication

The devices were designed using the Clewin Layout editor and ordered as film photomasks (JD Photo Data). Microfabrication was carried out on a 4‐inch silicon wafer, which was sonicated sequentially in acetone followed by isopropanol and dried thoroughly on a hot plate. A 2 µm layer of parylene‐C was deposited onto the silicon wafer via chemical vapor deposition (CVD, Speciality Coating Systems Labcoater, USA). An image‐reversal photoresist (AZ 5214E, Microchemicals GmbH, Germany) defined the electrode and interconnect geometry through photolithography (SUSS MABA6, Mask Aligner) and developed in an aqueous dilution of AZ351B at a ratio of 1:4 (Microchemicals GmbH, Germany). This was followed by electron beam evaporation of the metal electrodes and interconnects (Ti, 10‐nm adhesion layer and Au, 100 nm; PVD 75, Kurt J Lesker) and acetone lift‐off. The PEDOT:PSS solution was prepared by mixing a PEDOT:PSS suspension (Clevios PH 1000, Heraeus, Germany), 5% (v/v) ethylene glycol, and approximately 30 µL dodecyl benzene sulfonic acid (Sigma Aldrich, UK). A cross‐linker, 1% (v/v) 3‐Glycidyloxypropyltrimethoxysilane (GOPS, Sigma–Aldrich, UK), was added and filtered through a 0.45 µm pore‐size poly(tetrafluoroethylene) filter (Sartorius). This mixture was used within 20 min, by spin‐coating in 4 layers onto the silicon wafer to create a 600 nm thick layer. The sample was baked for 1 minute at 110°C between each coating step, and at 120°C for 1 h after the four layers were coated. Overnight soaking in deionized water was performed to remove excess PSS and low‐molecular‐weight compounds. The electrodes were further defined by photolithographic patterning of PEDOT:PSS using a positive photoresist (AZ5214E) developed in the aqueous dilution of the developer AZ351B at a ratio of 1:4 (Microchemicals GmbH, Germany), and reactive ion etching of PEDOT:PSS (CF¬4, 50 sccm, SF¬6, 20 sccm, 30 mTorr, 200W; PlasmaPro RIE 80, Oxford Instruments, UK). Finally, the wafer surface was plasma‐activated and immersed in a self‐assembled monolayer (SAM) of the adhesion promoter 3% (v/v) methacryloxypropyl trimethoxysilane (A174 silane, Sigma Aldrich, UK) prepared in 96% ethanol (containing 1% acetic acid) for 30 s followed by an ethanol rinse and heat treatment on a hot plate at 70°C for 1 h. This enhanced the adhesion of the subsequently deposited 2 µm thin parylene‐C encapsulation layer (chemical vapor deposition, Speciality Coating Systems Labcoater, USA). Finally, two separate patterning steps were performed to define the device outline and the electrode openings, using a positive photoresist (AZ10XT) in conjunction with AZ 726 MIF. Etching for each patterning step was performed using a reactive ion etcher (CF4, 8 sccm; SF6, 2 sccm; O2, 50 sccm; 60 mTorr) (PlasmaPro RIE 80 from Oxford Instruments, UK). The flexible devices were gently peeled from the silicon wafer, soaked in deionized water, transferred onto glass slides using a paint brush, and dried on an 80°C hot plate.

### 4.2 Device Release and Cable Bonding

Jumper cables (FFC/FPC, Molex 15014‐0613) were bonded using an anisotropic conductive adhesive film (ACF) (TGP5010 with 10 µm particle size, Telephus Inc., Korea) and a die bonder (FINEPLACER pico2, Finetech GmbH, Germany). The bonded probes were subsequently released from the glass slides using deionized water, and a low‐toxicity, fast‐curing silicone adhesive (Kwik SilTM) was used to seal the bonded interconnects.

### 4.3 Electrochemical Impedance Spectroscopy

Electrochemical Impedance Spectroscopy was performed with a potentiostat (Autolab Potentiostat, Metrohm AG, Switzerland) using a platinum (Pt) electrode as the counter electrode and a silver‐silver chloride (Ag/AgCl) reference electrode. A 10‐mV amplitude sinusoidal voltage input with frequencies ranging from 1 Hz to 100 kHz was used. The impedance of PEDOT:PSS was additionally measured using an Intan RHS stim/recording system (Intan Technologies, Los Angeles, CA) in 1x phosphate‐buffered saline (PBS) at 1 kHz.

### 4.4 Scanning Electron Microscope (SEM) Imaging

SEM images were taken using a ZEISS Gemini 300 VP scanning electron microscope with an acceleration voltage of 15 kV in the backscattered electron mode. The flexible device mounted on a microscopy slide was placed on top of the carbon tape and analyzed in point‐by‐point scanning mode.

### 4.5 Human Stem Cell, Organoid, and ALI‐CO Culture

H9 (WA09, RRID: CVCL_9773) embryonic stem cells purchased from WiCell (2015) were approved for use in this study by the UK Stem Cell Bank Steering Committee and local institutional ethics and health and safety reviews. Cell lines were confirmed for their identity by HTA profiling and regularly tested for mycoplasma contamination as well as cultured in the absence of antibiotics to ensure contamination free cultures. Cells were maintained in StemFlex medium (ThermoFisher Scientific, A3349401) on plates coated with Matrigel (Corning, 356234) and passaged twice weekly with 0.1M EDTA. The commercially available STEMdiff Cerebral Organoid Kit (StemCell Technologies, 08570) was used to generate cerebral organoids by seeding 2000 cells per embryoid body. After 15 days in vitro (DIV), the cerebral organoids were excised from the Matrigel droplets,] in which they were embedded during the kit protocol, with a needle and fine scalpel under a stereomicroscope and returned to organoid media. After DIV 30, the organoid medium was continually supplemented with Matrigel dissolved in 2% (v/v). For culture at the air–liquid interface (ALI), cerebral organoids (CO) of 55–70 (DIV) were processed into 300 µm slices as previously described [14] and collected directly onto cell culture inserts (Millipore, PICMORG50) with serum‐free slice culture medium beneath (SFSCM: Neurobasal (ThermoFisher Scientific, 21103049), 1x B‐27 supplement (ThermoFisher Scientific, 17504044), 1x Glutamax (ThermoFisher Scientific, 35050038), 0.5% (w/v) glucose). After two weeks of ALI‐CO culture, the media was switched to BrainPhys Neuronal Medium with N2‐A and SM1 (StemCell Technologies, 05793), supplemented with 35ng/ml vitamin C. All ALI‐CO media were supplemented with 1x antibiotic‐antimycotic (ThermoFisher Scientific, 15240062) and an additional 1:1000 (v/v) fungizone (Merck, A2942), and the medium was refreshed daily. All cell, organoid, and ALI‐CO cultures were maintained at 5% CO_2_ and 37°C. To account for common heterogeneity in organoid cultures, the presented data have been acquired across independent differentiation batches.

### 4.6 Design and Assembly of 3D Printed Culture Platform

The 3D printed culture platform was designed in OnShape and comprises three components: a cell culture insert adapter, a holder, and a PCB plug‐in module. All parts were 3D printed using polycarbonate filament and designed to withstand autoclaving and fit standard 35 mm cell culture dishes. Before first use, components were washed with soapy water and air‐dried. For assembly, the cell culture inserts (Millipore, PICMORG50), 3D‐printed parts, and Neuroweb were rinsed with 70% ethanol and air‐dried. The insert was assembled into the adapter, and press‐fit into the cell culture dish (ThermoFisher Scientific). Prior to plating ALI‐COs slices, the inserts and Neuroweb were washed once with PBS and three times with ALI‐CO media. Neuroweb was placed centrally onto the surface of the insert, with the FFC cable carefully secured in its slot, and flattened with a fine paintbrush wetted with culture media water to promote adhesion and conformational contact between the device and the membrane via surface tension. This interfacial media layer further ensured the preservation of a stable electrode impedance and consistent electrical coupling. ALI‐CO slices were then collected onto the inserts and positioned centrally on Neuroweb. Gentle flooding of the top surface of the insert with ALI‐CO media allowed ALI‐COs to float for further manipulation and reorientation to achieve optimal positioning before removal of the excess media on the insert.

### 4.7 ALI‐CO Fixation and Immunohistochemistry

ALI‐COs were fixed in 4% PFA overnight at 4*°*C and subsequently washed three times in PBS. Whole ALI‐COs were stained as previously described [14] with all steps performed in PBS with 0.25% Triton‐X and 4% normal donkey serum. The primary antibodies used were: GFAP (Abcam, ab7260, 1:1000 dilution), MAP2 (Abcam, ab5392, 1:200 dilution), and SMI312 (Biolegend, #837904, 1:500 dilution). Secondary antibodies (Thermo Fisher Scientific and Abcam) with AlexaFluor 488‐, 568‐, and 647‐ labels were used for detection. Imaging of stained whole ALI‐COs was performed using a Nikon CSU‐W1 spinning disk microscope with a 10x air‐ or 25x silicon‐immersion lens. All antibodies used were well‐described and tested in literature. No antibodies were novel or unvalidated according to the manufacturer.

### 4.8 Live/Dead Staining and Imaging

Single‐plane confocal images (10X objective) of DIV 200 ALI‐COs derived from H9 human embryonic stem cells and stained with the LIVE/DEAD Viability/Cytotoxicity Kit (ThermoFisher, L3224) were acquired on a Zeiss LSM 880. The MEA‐mounted slices was transferred onto the flexible PEDOT:PSS‐based MEA device on DIV 141 and maintained on‐device for 59 days of culture, while the control organoids were maintained on a standard air–liquid interface throughout. Organoid slices obtained from different source organoids were allocated randomly. Calcein‐AM (2 µM, green) labels live cells with intact intracellular esterase activity and ethidium homodimer‐1 (4 µM, red) labels cells with compromised plasma membranes. Organoids were incubated with the staining solution in PBS for 1 h at 37°C, then washed three times in PBS prior to imaging.

### 4.9 RNA‐sequencing (RNA‐seq) and Analysis

RNA extraction for all samples was conducted in parallel using the Direct‐zol RNA extraction kit for 96 samples (Zymo Research, R2054), as to the manufacturer's instruction. Poly(A) mRNA transcripts were isolated, and cDNA libraries were generated using NEBNext modules for library preparations (New England Biolabs, E7490 and E7760), according to the manufacturer's instructions, during which unique dual‐index UMI adapters (oligos for multiplexing sequencing) were incorporated (New England Biolabs, E7416 and E7874). Libraries were sequenced using NextSeq 2000 with a P3 flow cell and cartridge.

Bulk RNA sequencing data were aligned to the genome assemblies using the NextFlow RNA‐seq pipeline (version 3.12.0; nf‐co.re/rnaseq/3.12.0/) [73]. Data were aligned to the Homo sapiens assembly GRCh38.p14 (GCA_000001405.29; release 102) and alignment employed the default RNA‐seq pipeline parameters to output values expressed as transcripts per million (TPM).

Single‐cell data from ALI‐COs were obtained from Giandomenico et al. (2019) [14] and were reanalyzed starting from the gene expression matrix using Seurat version 5.1.0 in R (version 4.4.0). Cells were filtered for healthy cells with less than 1.8% mitochondrial reads and nCount_RNA >2100 but less than 3000 and nFeature_RNA >800 but less than 1500 to exclude multiplets after exploring the nFeature_RNA × nCount_RNA graph. Data normalisation, variable features, and scaling were done according to the standard Seurat pipeline. Clustering was done with a resolution of 0.43, and U‐MAP visualization was performed on the top 50 principal components. Clusters were annotated based on the top 20 differentially expressed genes according to log2FC, guided by known key marker genes.

For comparison with single‐cell RNA‐seq (scRNA‐seq) and bulk RNA‐seq analysis, the TPM matrix was first filtered for highly expressed genes with TPM greater than or equal to 1 and then log‐transformed for comparison with scRNA‐seq. Clusters from scRNA‐seq were used to generate pseudobulk data by first sub‐setting the Seurat object by each cluster, followed by conversion to a sum gene expression matrix of the raw counts and calculation of the counts per million (CPM). Only genes with a CPM of at least 1 in one sample were filtered, and the data were log‐transformed and merged with the bulk TPM matrix. The top 2000 most variable genes were then visualized by Pearson correlation using heatmap.2 and PCA analysis using prcomp. Deconvolution was performed using the bioconductor package, Granulator version 1.12.0.

### 4.10 Acute Rat Brain Slices

Acute rat slices were prepared from freshly dissected opportunistic rat brain provided by the institutional biological services facility and according to the institutional Animal Welfare Ethical Review Board and Home Office Approvals.

### 4.11 Electrophysiological Recording

A custom‐made printed‐circuit board was connected to the FFC/FPC cable of the device using a zero‐insertion force (ZIF) connector (FH39 series, Hirose) on one side and the RHS 32‐channel head‐stage with the RHS2116 amplifier head‐stage on the other side using an Omnetics connector (NPD‐36‐AA‐GS, Omnetics). The amplifier head‐stage was subsequently connected to the Intan RHS recording/stim system using a serial peripheral interface (SPI) cable. The device was designed to encompass an internal reference and ground, as reflected in the PCB layout. The sampling rate of the recording was 30 kHz, and the recording was performed in a grounded setup in an incubator, which acted as a Faraday cage. An Arduino MEGA was also connected to the Intan RHS recording/stim setup and was used to trigger recordings every hour for 5 min.

### 4.12 Data Pre‐Processing–Data Loading

For single file analysis, MEA data (.rhs and .rhd) was loaded onto NeuroMaps to extract the embedded information, such as the recorded signals, timestamps, channel identifiers, impedance values (optional), and phase information (optional). For batch analysis, the data saved with identifiers such as ‘experimental ID’, ‘spikeData’, ‘lfpData’ was loaded into the ‘aggregate_analysis.m’ NeuroMaps module for longitudinal data analysis.

### 4.13 Data Pre‐Processing–Quality Assessment

Electrodes were first screened based on impedance measurements, and any channel with impedance exceeding 1MΩ was excluded to eliminate non‐functional or unstable electrodes. In the case where impedance measurements were not collected, this step was skipped. Following this hardware‐level filtering, additional statistical and spectral metrics were used to identify noisy or artefactual channels. Channels were flagged if their standard deviation (STD) or median absolute deviation (MAD) exceeded 3.5 times the median across all channels, indicating abnormally high variance. Power spectral density (PSD) was then computed using Welch's method to quantify frequency‐domain characteristics of the signal. High‐frequency noise was evaluated by measuring the power above 80% of the Nyquist frequency. Channels exceeding a high‐frequency PSD threshold of 0.02 µV2/Hz were further assessed for coherence with the rest of the array. Any channel failing one or more of these criteria was excluded from downstream spike detection and analysis. This approach is based on methods developed by the International Brain Laboratory (IBL) [74] and previously applied by Spikeinterface [34].

### 4.14 Data Pre‐Processing–Referencing and Filtering

The data of the corresponding good channels was filtered for powerline hum using an infinite impulse response (IIR) filter of order 4 between 50 Hz and 6 kHz at 100 Hz increments (harmonics) with a 2 Hz bandwidth around each harmonic. The cleaned data was subsequently referenced using a common median reference (CMR) rather than a common average reference (CAR). This method is preferred to avoid data biasing during high spiking activity [6] Referenced data was then band‐pass filtered (3rd order Butterworth filter, 100–6000 Hz) to detect multi‐unit activity. For low‐frequency analysis, the powerline filtered data was down‐sampled to 1 kHz and low‐pass filtered using a bi‐directional finite impulse response filter (FIR) using eegfilt.m (EEGLAB) [75]. This was done at 500 Hz and at the sub‐band frequencies previously reported6: delta (0.5–4 Hz), theta (4–7 Hz), alpha (8–13 Hz), beta (13–30 Hz), gamma (30–50 Hz) and broadband high gamma (100–400 Hz). Similar to previous work, high gamma analysis was performed in separate segments, 100–200 and 200–400 Hz, to prevent biasing the power estimates toward lower frequency limits due to the inverse power law (1/f) that the power spectrum follows.

### 4.15 Multi‐Unit Activity (MUA) Analysis–Spike Detection

Spikes were detected for each channel using a 60‐s window and a threshold‐based method, in which the threshold was set to 4.5σ_n_. This value can be adjusted based on the noise levels of the signal visualized in NeuroMaps. The noise level was robustly estimated using the formula, σ_n_ = median(|S_t_|/0.6745), where |S_t_| is the absolute signal amplitude [45]. For spike detection, ‘findpeaks.m’ was used, where a peak was defined by its minimum peak height (4.5 σ_n_) and a minimum peak distance (3 ms). A maximum peak height was also set (30 σ_n_) to eliminate spurious charges. Duplicate spikes were removed by sorting the spike locations of the concatenated positive and negative spikes and removing the lower amplitude spike occurring within the refractory period (2 ms). Non‐physiological waveforms were also detected by identifying and eliminating instances where more than 3 spikes were detected within the refractory period. Moreover, spikes exceeding a full width at half maximum threshold of 1 ms were considered non‐biological and were excluded from further analysis. The spikes were centered around a 4 ms window.

### 4.16 MUA Analysis–Spike Sorting and Clustering

Preliminary clustering was performed as a general quality check of waveform separability. Waveforms were first z‐score normalized, reduced in dimensionality using principal component analysis (PCA), and clustered using k‐means with a minimum cluster size of 10. Mean and standard deviation waveforms were visualized for each cluster, and non‐physiological events were discarded.

For more refined sorting, we applied a previously published method integrating wavelet decomposition with Gaussian mixture modelling (GMM) [76]. Briefly, waveforms were decomposed using a Haar wavelet transform, and the resulting coefficients were z‐score normalized. Each wavelet component was weighted by a GMM‐based separability metric prior to PCA. The top five principal components were retained, and up to 12 Gaussians were used to overfit the data in high‐dimensional space. Final cluster centroids were refined using Nelder–Mead optimization to identify local density maxima in feature space, and distinct spike clusters were accordingly labeled [77].

Following clustering, spike timestamps were used to generate a binary vector matching the length of the original signal, with ‘1’ indicating a spike event and ‘0’ elsewhere. This binary array was used to generate raster plots and in downstream population activity analyses.

### 4.17 MUA Analysis–Network Analysis

Population network activity was visualized by summing the binary spike arrays across all electrodes in 1 ms bins. The resulting population firing rate was smoothed using a 100 ms Gaussian kernel to reveal underlying network dynamics. Bin size and smoothing window were selected based on previously reported methods and parameters to preserve temporal resolution while reducing noise [6, 31]. Functional connections were identified using spike‐time‐tiling‐correlation matrix (STTC) [47] and the nodes were classified as receiver, sender, or broker nodes by computing the latency in spikes detected at each electrode and comparing the incoming and outgoing number of spikes such that they exceed 80%.

### 4.18 MUA Analysis–Spike Features and Synchronicity

Various features including full‐width half maximum, peak‐to‐peak amplitude, inter‐spike‐interval (ISI), and bursts. Bursts were detected if they had a minimum duration of 50 ms and contained a minimum of 3 spikes with a maximum ISI of 100 ms. The synchronicity was evaluated in the case where more than 8 channels were active and computed using SpikeContrast [78].

### 4.19 Local‐Field Potential (LFP) Analysis–Oscillatory Analysis

To quantify oscillatory activity independently from aperiodic (1/f) components, power spectral densities (PSDs) were computed using Welch's method (2 s window with 50% overlap). Prior to using the fitting oscillations and one‐over‐f (FOOOF) toolbox32, the PSDs were interpolated at frequencies affected by powerline notch filtering (50 Hz and its harmonics) to smooth the dips which might confound the results. Subsequently, log power spectra were fit between 1 and 100 Hz. The FOOOF model separates the power spectrum into an aperiodic component (modelled as a linear function in log‐log space) and a set of periodic peaks.

The aperiodic exponent and offset were extracted as measures of broadband activity. Oscillatory power was assessed by summing the power of identified peaks after subtracting the aperiodic fit. Only peaks with power above a defined threshold and goodness‐of‐fit exceeding R2>0.9 were retained. Parameters for FOOOF were based on previous work [32].

### 4.20 LFP Analysis–Phase Amplitude Coupling (PAC)

To assess PAC, each filtered sub‐band was processed using a Hilbert transform to extract the instantaneous phase and amplitude. The phase spanning −π to π was then discretized into 20 bins, π/10 each. Corresponding amplitude values (i.e., power) were grouped by phase bin, and visualized by circularly shifting the distribution so that the peak occurred at −π similar to previous work [6].

### 4.21 LPF Analysis–Modulation Index (MI)

The strength of the phase‐amplitude coupling strength was quantified using the modulation index, which is based on the Kullback–Leibler (KL) divergence [37]. This metric captures the deviation of the amplitude distribution across the phase bins from a uniform distribution, indicating the degree of coupling.

### 4.22 LFP Analysis–Continuous Wavelet Transform (CWT)

Continuous wavelet transform was used to decompose the low‐pass filtered neuronal signal into specific frequency bands to obtain time‐frequency signal representations while preserving both spectral and temporal information.

### 4.23 Functionality Testing

The brain organoids were recorded in BrainPhys medium. For functionality testing, 50 mM potassium chloride (KCl) was used to modify the electrical activity of the organoids. Baseline measurements were obtained before and after the reagent addition. The sample was rinsed three times with Dulbecco's phosphate‐buffered saline (DPBS) and fresh medium was added after each reagent treatment.

For additional functionality testing using TTX, bicuculline, and DMSO control treatments, the extracellular electrophysiological recordings were analyzed in MATLAB using NeuroMaps, while the acute recordings were acquired via established planar PEDOT:PSS‐based microelectrode arrays to ensure biological relevance [23]. Specifically, extracellular activity was recorded from cerebral organoids (DIV 140). Treatment conditions were as follows: the voltage‐gated sodium channel blocker tetrodotoxin (TTX; 2 µM), the GABA‐A receptor antagonist bicuculline (10 µM), or 0.1% DMSO vehicle control. For each organoid, recordings were acquired at three timepoints: immediately prior to treatment (baseline), 15 min after treatment application, and 2 h after treatment following PBS washouts. Signals were recorded at 25 000 Hz, before being band‐pass filtered from 300–6000 Hz using a third‐order Butterworth filter. Positive and negative polarity spikes were detected using an amplitude threshold of 4.0 standard deviation relative to noise (as previously described). Detected events with peak‐to‐peak amplitude below 10 µV were removed from downstream analyses. Each data point represents the mean from one organoid.

### 4.24 Statistical Analysis

All statistical analyses were performed in GraphPad Prism 8, with the specific tests and sample sizes provided in the corresponding figure captions. *p<0.05, **p<0.01, ***p<0.001, and ****p<0.0001, while non‐significant comparisons are denoted as n.s.

## Funding

G.S.K.S. acknowledges funding from the Wellcome Trust (065807/Z/01/Z) (203249/Z/16/Z), the UK Medical Research Council (MRC) (MR/K02292X/1), Alzheimer Research UK (ARUK), (ARUK‐PG013‐14), Michael J Fox Foundation (16238 and 022159), and Infinitus China Ltd. N.F.L acknowledges funding from the Swiss National Science Foundation (P2EZP2_19984), and B.H., S.M., S.V., R.N.K. acknowledge funding from the Cambridge Trust. B.H. was also supported by a Trinity Henry Barlow Scholarship, R.N.K. by a King's College Cambridge Scholarship, and S.V. by an EPSRC Doctoral Training Programme (G115857). M.A.L. acknowledges funding from the UK Medical Research Council (MC_UP_1201/9). S.M., J.S.R., and R.R.M were supported by EPSRC (EP/L015889/1; EP/S022139/1; EP/L015978/1), and J.T. by a Gates Cambridge Scholarship.

## Conflicts of Interest

Cambridge Enterprise has filed a patent application that covers the device, system, and methods of NeuroSuite (GB 2511343.2, listing B.H., S.M., D.J.L.D.S., N.F.L., S.V., R.N.K., M.A.L., G.M.M., and G.S.K. as owners). M.A.L. is an inventor on patents covering cerebral organoids and is founder and advisory board member of a:head bio. The other authors declare no competing interests.

## Supporting information




**Supporting File**: advs76956‐sup‐0001‐SuppMat.pdf.

## Data Availability

The custom code for NeuroMaps is available on Github (https://github.com/BelNeuroCoding/NeuroMaps) and Zenodo (10.5281/zenodo.19120269). Code and data associated with bulk and scRNA‐seq analysis have are available on Github (https://github.com/madlancaster/Neuroweb). Further data is available from the corresponding author upon reasonable request.

## References

[1] M. E. Spira and A. Hai , “Multi‐electrode Array Technologies for Neuroscience and Cardiology,” Nature Nanotechnology 8, no. 2 (2013): 83–94. 10.1038/nnano.2012.265 23380931

[2] M. A. Lancaster , M. Renner , C.‐A. Martin , et al., “Cerebral Organoids Model human Brain Development and Microcephaly,” Nature 501, no. 7467 (2013): 373–379. 23995685 10.1038/nature12517 PMC3817409

[3] I. Kelava and M. A. Lancaster , “Stem Cell Models of Human Brain Development,” Cell Stem Cell 18, no. 6 (2016): 736–748. 27257762 10.1016/j.stem.2016.05.022

[4] E. Di Lullo and A. R. Kriegstein , “The Use of Brain Organoids to Investigate Neural Development and Disease,” Nature Reviews Neuroscience 18 (2017): 573–584. 28878372 10.1038/nrn.2017.107 PMC5667942

[5] F. R. Cugola , I. R. Fernandes , F. B. Russo , et al., “The Brazilian Zika Virus Strain Causes Birth Defects in Experimental Models,” Nature 534, no. 7606 (2016): 267–271. 27279226 10.1038/nature18296 PMC4902174

[6] C. A. Trujillo , R. Gao , P. D. Negraes , et al., “Complex Oscillatory Waves Emerging from Cortical Organoids Model Early Human Brain Network Development,” Cell Stem Cell 25, no. 4 (2019): 558–569.e7. 31474560 10.1016/j.stem.2019.08.002 PMC6778040

[7] H. Wang , J. A. Kulas , C. Wang , D. M. Holtzman , H. A. Ferris , and S. B. Hansen , “Regulation of Beta‐amyloid Production in Neurons by Astrocyte‐derived Cholesterol,” Proceedings of the National Academy of Sciences 118, no. 33 (2021): 2102191118. 10.1073/pnas.2102191118 PMC8379952 34385305

[8] P. Conforti , D. Besusso , V. D. Bocchi , et al., “Faulty Neuronal Determination and Cell Polarization Are Reverted by Modulating HD Early Phenotypes,” Proceedings of the National Academy of Sciences 115, no. 4 (2018): E762–E771. 10.1073/pnas.1715865115 PMC5789931 29311338

[9] B. R. Groveman , S. T. Foliaki , C. D. Orru , et al., “Sporadic Creutzfeldt‐Jakob Disease Prion Infection of human Cerebral Organoids,” Acta Neuropathologica Communications 7, no. 1 (2019): 90. 31196223 10.1186/s40478-019-0742-2 PMC6567389

[10] T. Matsui and T. Shinozawa , “Human Organoids for Predictive Toxicology Research and Drug Development,” Frontiers in Genetics 12 (2021): 767621. 34790228 10.3389/fgene.2021.767621 PMC8591288

[11] T. Chhibber , S. Bagchi , B. Lahooti , et al., “CNS Organoids: an Innovative Tool for Neurological Disease Modeling and Drug Neurotoxicity Screening,” Drug Discovery Today 25, no. 2 (2020): 456–465. 31783130 10.1016/j.drudis.2019.11.010 PMC7039749

[12] T. Hartung , “Thoughts on Limitations of Animal Models,” Parkinsonism & Related Disorders 14 (2008): S81–S83. 18585949 10.1016/j.parkreldis.2008.04.003

[13] M. Geiger , Fundamentals of Vascular Biology (Springer, 2019).

[14] S. L. Giandomenico , S. B. Mierau , G. M. Gibbons , et al., “Cerebral Organoids at the Air–liquid Interface Generate Diverse Nerve Tracts with Functional Output,” Nature Neuroscience 22, no. 4 (2019): 669–679. 30886407 10.1038/s41593-019-0350-2 PMC6436729

[15] B. H. Gähwiler , M. Capogna , D. Debanne , R. A. McKinney , and S. M. Thompson , “Organotypic Slice Cultures: a Technique Has Come of Age,” Trends in Neurosciences 20, no. 10 (1997): 471–477. 9347615 10.1016/s0166-2236(97)01122-3

[16] L. A. Mulder , J. A. Depla , A. Sridhar , K. Wolthers , D. Pajkrt , and R. Vieira de Sá , “A Beginner's Guide on the Use of Brain Organoids for Neuroscientists: A Systematic Review,” Stem Cell Research & Therapy 14, no. 1 (2023): 1–21. 37061699 10.1186/s13287-023-03302-x PMC10105545

[17] C. Sun , Z. Cheng , J. Abu‐Halimah , and B. Tian , “Perspectives on Tissue‐like Bioelectronics for Neural Modulation,” Iscience 26, no. 5 (2023): 106715. 37216128 10.1016/j.isci.2023.106715 PMC10192532

[18] J. S. Choi , H. J. Lee , S. Rajaraman , and D. H. Kim , “Recent Advances in Three‐dimensional Microelectrode Array Technologies for in Vitro and in Vivo Cardiac and Neuronal Interfaces,” Biosensors and Bioelectronics 171 (2021): 112687. 33059168 10.1016/j.bios.2020.112687 PMC7665982

[19] P. D. Jones , T. Stumpp , M. Mierzejewski , D. Pascual , and A. Stumpf , “Scalable Mesh Microelectrode Arrays for Neural Spheroids and Organoids,” Current Directions in Biomedical Engineering 9 (2023): 575–578.

[20] K. Tasnim and J. Liu , “Emerging Bioelectronics for Brain Organoid Electrophysiology,” Journal of Molecular Biology 434, no. 3 (2022): 167165. 34293341 10.1016/j.jmb.2021.167165 PMC8766612

[21] A. P. Passaro and S. L. Stice , “Electrophysiological Analysis of Brain Organoids: Current Approaches and Advancements,” Frontiers in Neuroscience 14 (2021): 622137. 33510616 10.3389/fnins.2020.622137 PMC7835643

[22] W. G. Chung , E. Kim , H. Song , et al., “Recent Advances in Electrophysiological Recording Platforms for Brain and Heart Organoids,” Advanced NanoBiomed Research 2, no. 12 (2022): 2200081.

[23] S. Middya , V. F. Curto , A. Fernández‐Villegas , et al., “Microelectrode Arrays for Simultaneous Electrophysiology and Advanced Optical Microscopy,” Advanced Science 8, no. 13 (2021): 2004434. 36246164 10.1002/advs.202004434 PMC9539726

[24] E. Fernández , et al., “Acute Human Brain Responses to Intracortical Microelectrode Arrays: Challenges and Future Prospects,” Front Neuroeng 7 (2014): 24. 25100989 10.3389/fneng.2014.00024 PMC4104831

[25] L. Jiang , et al., “A Review on Mechanical Considerations for Chronically‐Implanted Neural Probes,” Journal of Neural Engineering 15 (2018): 031001. 28885187 10.1088/1741-2552/aa8b4f

[26] G. Hong and C. M. Lieber , “Novel Electrode Technologies for Neural Recordings,” Nature Reviews Neuroscience 20, no. 6 (2019): 330–345. 30833706 10.1038/s41583-019-0140-6 PMC6531316

[27] Y. Park , C. K. Franz , H. Ryu , et al., “Three‐dimensional, Multifunctional Neural Interfaces for Cortical Spheroids and Engineered Assembloids,” Science Advances 7, no. 12 (2021): 9153–9170. 10.1126/sciadv.abf9153 PMC7968849 33731359

[28] T. L. Li , Y. Liu , C. Forro , et al., “Stretchable Mesh Microelectronics for the Biointegration and Stimulation of human Neural Organoids,” Biomaterials 290 (2022): 121825. 36326509 10.1016/j.biomaterials.2022.121825 PMC9879137

[29] Q. Huang , B. Tang , J. C. Romero , et al., “Shell Microelectrode Arrays (MEAs) for Brain Organoids,” Science Advances 8, no. 33 (2022): 5031. 10.1126/sciadv.abq5031 PMC9385157 35977026

[30] P. Le Floch , et al., “Stretchable Mesh Nanoelectronics for 3D Single‐Cell Chronic Electrophysiology from Developing Brain Organoids,” Advanced Materials 34 (2022): 2106829. 10.1002/adma.202106829 PMC8930507 35014735

[31] X. Yang , C. Forró , T. L. Li , et al., “Kirigami Electronics for Long‐term Electrophysiological Recording of human Neural Organoids and Assembloids,” Nature Biotechnology 42, no. 12 (2024): 1836–1843. 10.1038/s41587-023-02081-3 PMC11260907 38253880

[32] T. Donoghue , M. Haller , E. J. Peterson , et al., “Parameterizing Neural Power Spectra into Periodic and Aperiodic Components,” Nature Neuroscience 23, no. 12 (2020): 1655–1665. 33230329 10.1038/s41593-020-00744-x PMC8106550

[33] J. Bobacka , A. Lewenstam , and A. Ivaska , “Electrochemical Impedance Spectroscopy of Oxidized Poly(3,4‐ethylenedioxythiophene) Film Electrodes in Aqueous Solutions,” Journal of Electroanalytical Chemistry 489, no. 1‐2 (2000): 17–27.

[34] A. P. Buccino , C. L. Hurwitz , S. Garcia , et al., “Spikeinterface, a Unified Framework for Spike Sorting,” Elife 9 (2020): 1–24. 10.7554/eLife.61834 PMC7704107 33170122

[35] D. Kireev , V. R. Montes , J. Stevanovic , K. Srikantharajah , and A. Offenhäusser , “N3‐MEA Probes: Scooping Neuronal Networks,” Frontiers in Neuroscience 13 (2019): 442809. 10.3389/fnins.2019.00320 PMC6467947 31024239

[36] D. A. Soscia , D. Lam , A. C. Tooker , et al., “A Flexible 3‐dimensional Microelectrode Array for in Vitro Brain Models,” Lab on a Chip 20, no. 5 (2020): 901–911. 31976505 10.1039/c9lc01148j

[37] A. B. L. Tort , R. Komorowski , H. Eichenbaum , and N. Kopell , “Measuring Phase‐amplitude Coupling between Neuronal Oscillations of Different Frequencies,” Journal of Neurophysiology 104, no. 2 (2010): 1195–1210. 20463205 10.1152/jn.00106.2010 PMC2941206

[38] J. H. Park , D. Y. Lee , Y.‐H. Kim , et al., “Flexible and Transparent Metallic Grid Electrodes Prepared by Evaporative Assembly,” ACS Applied Materials & Interfaces 6, no. 15 (2014): 12380–12387. 24999517 10.1021/am502233y

[39] M. O. Heuschkel , M. Fejtl , M. Raggenbass , D. Bertrand , and P. Renaud , “A Three‐dimensional Multi‐electrode Array for Multi‐site Stimulation and Recording in Acute Brain Slices,” Journal of Neuroscience Methods 114, no. 2 (2002): 135–148. 11856564 10.1016/s0165-0270(01)00514-3

[40] J. Scholvin , A. Zorzos , J. Kinney , et al., “Scalable, Modular Three‐Dimensional Silicon Microelectrode Assembly via Electroless Plating,” Micromachines 9, no. 9 (2018): 436. 30424369 10.3390/mi9090436 PMC6187301

[41] N. Kunori and I. Takashima , “A Transparent Epidural Electrode Array for Use in Conjunction with Optical Imaging,” Journal of Neuroscience Methods 251 (2015): 130–137. 26049111 10.1016/j.jneumeth.2015.05.018

[42] P. Ledochowitsch , E. Olivero , T. Blanche , and M. M. Maharbiz , “A Transparent ECoG Array for Simultaneous Recording and Optogenetic Stimulation,” in Proceedings of the Annual International Conference of the IEEE Engineering in Medicine and Biology Society (IEEE, 2011): 2937–2940, 10.1109/IEMBS.2011.6090808. 22254956

[43] Y. Qiang , P. Artoni , K. J. Seo , et al., “Transparent Arrays of Bilayer‐nanomesh Microelectrodes for Simultaneous Electrophysiology and Two‐photon Imaging in the Brain,” Science Advances 4, no. 9 (2018): aat0626. 10.1126/sciadv.aat0626 PMC6124910 30191176

[44] Y. T. Lo , L. Jiang , B. Woodington , et al., “Recording of Single‐unit Activities with Flexible Micro‐electrocorticographic Array in Rats for Decoding of Whole‐body Navigation,” Journal of Neural Engineering 21, no. 4 (2024): 046037. 10.1088/1741-2552/ad618c 38986465

[45] H. G. Rey , C. Pedreira , and R. Quian Quiroga , “Past, Present and Future of Spike Sorting Techniques,” Brain Research Bulletin 119 (2015): 106–117. 25931392 10.1016/j.brainresbull.2015.04.007 PMC4674014

[46] M. S. Schroeter , P. Charlesworth , M. G. Kitzbichler , O. Paulsen , and E. T. Bullmore , “Emergence of Rich‐Club Topology and Coordinated Dynamics in Development of Hippocampal Functional Networks in Vitro,” The Journal of Neuroscience 35, no. 14 (2015): 5459–5470. 25855164 10.1523/JNEUROSCI.4259-14.2015 PMC4388914

[47] C. S. Cutts and X. S. J. Eglen , “Detecting Pairwise Correlations in Spike Trains: an Objective Comparison of Methods and Application to the Study of Retinal Waves,” The Journal of Neuroscience 34, no. 43 (2014): 14288–14303. 25339742 10.1523/JNEUROSCI.2767-14.2014 PMC4205553

[48] K. Kirmse and C. Zhang , “Principles of GABAergic Signaling in Developing Cortical Network Dynamics,” Cell Reports 38, no. 13 (2022): 110568. 35354036 10.1016/j.celrep.2022.110568

[49] D. D. Wang and A. R. Kriegstein , “Defining the Role of GABA in Cortical Development,” The Journal of Physiology 587, no. 9 (2009): 1873–1879. 19153158 10.1113/jphysiol.2008.167635 PMC2689328

[50] M.‐P. Zafeiriou , G. Bao , J. Hudson , et al., “Developmental GABA Polarity Switch and Neuronal Plasticity in Bioengineered Neuronal Organoids,” Nature Communications 11, no. 1 (2020): 1–12. 10.1038/s41467-020-17521-w PMC7391775 32728089

[51] K. D. A. Rienecker , R. G. Poston , and R. N. Saha , “Merits and Limitations of Studying Neuronal Depolarization‐Dependent Processes Using Elevated External Potassium,” ASN Neuro 12, no. 1 (2020): 1759091420974807. 33256465 10.1177/1759091420974807 PMC7711227

[52] G. T. Einevoll , C. Kayser , N. K. Logothetis , and S. Panzeri , “Modelling and Analysis of Local Field Potentials for Studying the Function of Cortical Circuits,” Nature Reviews Neuroscience 14, no. 11 (2013): 770–785. 24135696 10.1038/nrn3599

[53] G. Buzsáki , Rhythms of the Brain (Oxford Academic, 2006): 1–464, 10.1093/ACPROF:OSO/9780195301069.001.0001.

[54] B. Voytek and R. T. Knight , “Dynamic Network Communication as a Unifying Neural Basis for Cognition, Development, Aging, and Disease,” Biological Psychiatry 77, no. 12 (2015): 1089–1097. 26005114 10.1016/j.biopsych.2015.04.016 PMC4443259

[55] J. R. Manning , J. Jacobs , I. Fried , and M. J. Kahana , “Broadband Shifts in Local Field Potential Power Spectra Are Correlated with Single‐Neuron Spiking in Humans,” The Journal of Neuroscience 29, no. 43 (2009): 13613–13620. 19864573 10.1523/JNEUROSCI.2041-09.2009 PMC3001247

[56] K. J. Miller , E. C. Leuthardt , G. Schalk , et al., “Spectral Changes in Cortical Surface Potentials during Motor Movement,” The Journal of Neuroscience 27, no. 9 (2007): 2424–2432. 17329441 10.1523/JNEUROSCI.3886-06.2007 PMC6673496

[57] R. Mukamel , H. Gelbard , A. Arieli , U. Hasson , I. Fried , and R. Malach , “Coupling between Neuronal Firing, Field Potentials, and fMRI in Human Auditory Cortex,” Science 309, no. 5736 (1979): 951–954. 10.1126/science.1110913 16081741

[58] T. Osaki , T. Duenki , S. Y. A. Chow , et al., “Complex Activity and Short‐term Plasticity of human Cerebral Organoids Reciprocally Connected with Axons,” Nature Communications 15, no. 1 (2024): 1–13. 10.1038/s41467-024-46787-7 PMC11006899 38600094

[59] S. Samiee and S. Baillet , “Time‐resolved Phase‐amplitude Coupling in Neural Oscillations,” Neuroimage 159 (2017): 270–279. 28757194 10.1016/j.neuroimage.2017.07.051

[60] M. Durens , J. Nestor , M. Williams , et al., “High‐throughput Screening of human Induced Pluripotent Stem Cell‐derived Brain Organoids,” Journal of Neuroscience Methods 335 (2020): 108627. 32032714 10.1016/j.jneumeth.2020.108627

[61] T. Sharf , T. van der Molen , S. M. K. Glasauer , et al., “Functional Neuronal Circuitry and Oscillatory Dynamics in human Brain Organoids,” Nature Communications 13, no. 1 (2022): 1–20. 10.1038/s41467-022-32115-4 PMC9338020 35906223

[62] G. Quadrato , T. Nguyen , E. Z. Macosko , et al., “Cell Diversity and Network Dynamics in Photosensitive human Brain Organoids,” Nature 545, no. 7652 (2017): 48–53. 28445462 10.1038/nature22047 PMC5659341

[63] K. V. Nemani , K. L. Moodie , J. B. Brennick , A. Su , and B. Gimi , “In Vitro and in Vivo Evaluation of SU‐8 Biocompatibility,” Materials Science and Engineering: C 33, no. 7 (2013): 4453–4459. 23910365 10.1016/j.msec.2013.07.001 PMC3843949

[64] Z. Chen and J. B. Lee , “Biocompatibility of SU‐8 and Its Biomedical Device Applications,” Micromachines 12 (2021): 794. 34357204 10.3390/mi12070794 PMC8304786

[65] M. McDonald , D. Sebinger , L. Brauns , et al., “A Mesh Microelectrode Array for Non‐invasive Electrophysiology within Neural Organoids,” Biosensors and Bioelectronics 228 (2023): 115223. 36931193 10.1016/j.bios.2023.115223

[66] M. Lu , E. Hui , M. Brockhoff , et al., “Graphene Microelectrode Arrays, 4D Structured Illumination Microscopy, and a Machine Learning Spike Sorting Algorithm Permit the Analysis of Ultrastructural Neuronal Changes during Neuronal Signaling in a Model of Niemann–Pick Disease Type C,” Advanced Science 11, no. 44 (2024): 2402967. 39340823 10.1002/advs.202402967 PMC11600250

[67] G. Bounova and O. De Weck , “Overview of Metrics and Their Correlation Patterns for Multiple‐metric Topology Analysis on Heterogeneous Graph Ensembles,” Physical Review E 85, no. 1 (2012): 016117. 10.1103/PhysRevE.85.016117 22400635

[68] G. L. Gerstein and B. Mandelbrot , “Random Walk Models for the Spike Activity of a Single Neuron,” Biophysical Journal 4, no. 1 (1964): 41–68. 14104072 10.1016/s0006-3495(64)86768-0 PMC1367440

[69] W. J. Ma , J. M. Beck , P. E. Latham , and A. Pouget , “Bayesian Inference with Probabilistic Population Codes,” Nature Neuroscience 9, no. 11 (2006): 1432–1438. 17057707 10.1038/nn1790

[70] M. Ganji , A. Tanaka , V. Gilja , E. Halgren , and S. A. Dayeh , “Scaling Effects on the Electrochemical Stimulation Performance of Au, Pt, and PEDOT:PSS Electrocorticography Arrays,” Advanced Functional Materials 27 (2017): 1703019.

[71] S. F. Cogan , “Neural Stimulation and Recording Electrodes,” Annual Review of Biomedical Engineering 10, no. 1 (2008): 275–309. 10.1146/annurev.bioeng.10.061807.160518 18429704

[72] W. Ling , J. Yu , N. Ma , et al., “Flexible Electronics and Materials for Synchronized Stimulation and Monitoring in Multi‐Encephalic Regions,” Advanced Functional Materials 30, no. 32 (2020): 2002644.

[73] H. Patel , P. Ewels , A. Peltzer , et al., nf‐core/rnaseq: nf‐core/rnaseq v3.12.0 ‐ Osmium Octopus, Zenodo, Version 3.12.0 [Computer software] (2023), 10.5281/zenodo.7998767.

[74] International Brain Laboratory , K. Banga , J. Benson , J. Bhagat , et al., “Reproducibility of in Vivo Electrophysiological Measurements in Mice,” Elife 13 (2025): RP100840. 40354112 10.7554/eLife.100840 PMC12068871

[75] A. Delorme and S. Makeig , “EEGLAB: an Open Source Toolbox for Analysis of Single‐trial EEG Dynamics Including Independent Component Analysis,” Journal of Neuroscience Methods 134, no. 1 (2004): 9–21. 15102499 10.1016/j.jneumeth.2003.10.009

[76] B. C. Souza , V. Lopes‐dos‐Santos , J. Bacelo , and A. B. L. Tort , “Spike Sorting with Gaussian Mixture Models,” Scientific Reports 9, no. 1 (2019): 1–14. 30842459 10.1038/s41598-019-39986-6 PMC6403234

[77] J. C. Lagarias , J. A. Reeds , M. H. Wright , and P. E. Wright , “Convergence Properties of the Nelder–Mead Simplex Method in Low Dimensions,” SIAM Journal on Optimization 9, no. 1 (2006): 112–147, 10.1137/S1052623496303470.

[78] M. Ciba , T. Isomura , Y. Jimbo , A. Bahmer , and C. Thielemann , “Spike‐contrast: a Novel Time Scale Independent and Multivariate Measure of Spike Train Synchrony,” Journal of Neuroscience Methods 293 (2018): 136–143. 28935422 10.1016/j.jneumeth.2017.09.008

[79] Q. Li , K. Nan , P. Le Floch , et al., “Cyborg Organoids: Implantation of Nanoelectronics via Organogenesis for Tissue‐Wide Electrophysiology,” Nano Letters 19, no. 8 (2019): 5781–5789. 31347851 10.1021/acs.nanolett.9b02512

[80] A. Carnicer‐Lombarte , A. J. Boys , A. Güemes , et al., “Ultraconformable Cuff Implants for Long‐term Bidirectional Interfacing of Peripheral Nerves at Sub‐nerve Resolutions,” Nature Communications 15, no. 1 (2024): 1–14. 10.1038/s41467-024-51988-1 PMC11364531 39214981

