# Supplementary material for: NeuroSuite for Long‐Term Functional and Structural Studies of Air‐Liquid Interface Cerebral Organoids
Source: Adv Sci (Weinh). 2026 Aug 6:e19893. Online ahead of print. doi: 10.1002/advs.202519893 (PMC13444802; doi:10.1002/advs.202519893)
Supplement: Supplementary file 1 [file ADVS-9999-e19893-s001.pdf]

Supporting Information

NeuroSuite for Long-term Functional and Structural Studies of Air-Liquid Interface Cerebral Organoids

*Belquis Haider, Sagnik Middya, Daniel Lloyd-Davies-Sánchez, Nino F. Läubli, Sulay Vora, Jakob Träuble, Rohan N. Krajeski, Yuqing Feng, Daniel Duncko, Josiah S. Riley, Rubén Ruiz-Mateos Serrano, Ole Paulsen, Madeline Lancaster, George G. Malliaras, Gabriele S. Kaminski Schierle**

Text S1. Bending Stiffness Calculations

To estimate the bending stiffness of Neuroweb, we modelled it as a three-layer beam stack comprising parylene-C (top and bottom encapsulation layers) and a conductive gold layer. The PEDOT:PSS coating was not included in this calculation, as its mechanical stiffness significantly decreases in aqueous environments owing to swelling and ion uptake, making its contribution in physiological environments negligible.

For a homogeneous beam, the bending stiffness is given by:

$$D = \frac{Ewh^3}{12}$$

where E is the elastic modulus, w is the beam width, and h is the beam thickness.

For a multi-layer structure, the effective bending stiffness is described as follows[79]:

$$D = \frac{1}{12}\left(\frac{E_i}{1-\nu_i^2}\right)\left(w_i h_i^3 - w_m h_m^3\right) + \frac{1}{12}\left(\frac{E_m}{1-\nu_m^2}\right) w_m h_m^3$$

where:

- E_i = 1.13 GPa and ν_i = 0.4 are the elastic modulus and Poisson's ratio for parylene-C[80], respectively.
- E_m = 79 GPa and ν_i = 0.43 are the elastic modulus and Poisson's ratio of gold, respectively.
- w_i = 100 µm and h_i = 4 µm are the width and total thickness of the parylene-C, respectively.
- w_m = 20 µm and h_m = 100 nm are the width and total thickness of the conductive gold traces, respectively.

Substituting these values yields:

$$D \cong 0.72 \text{ pNm}^2$$

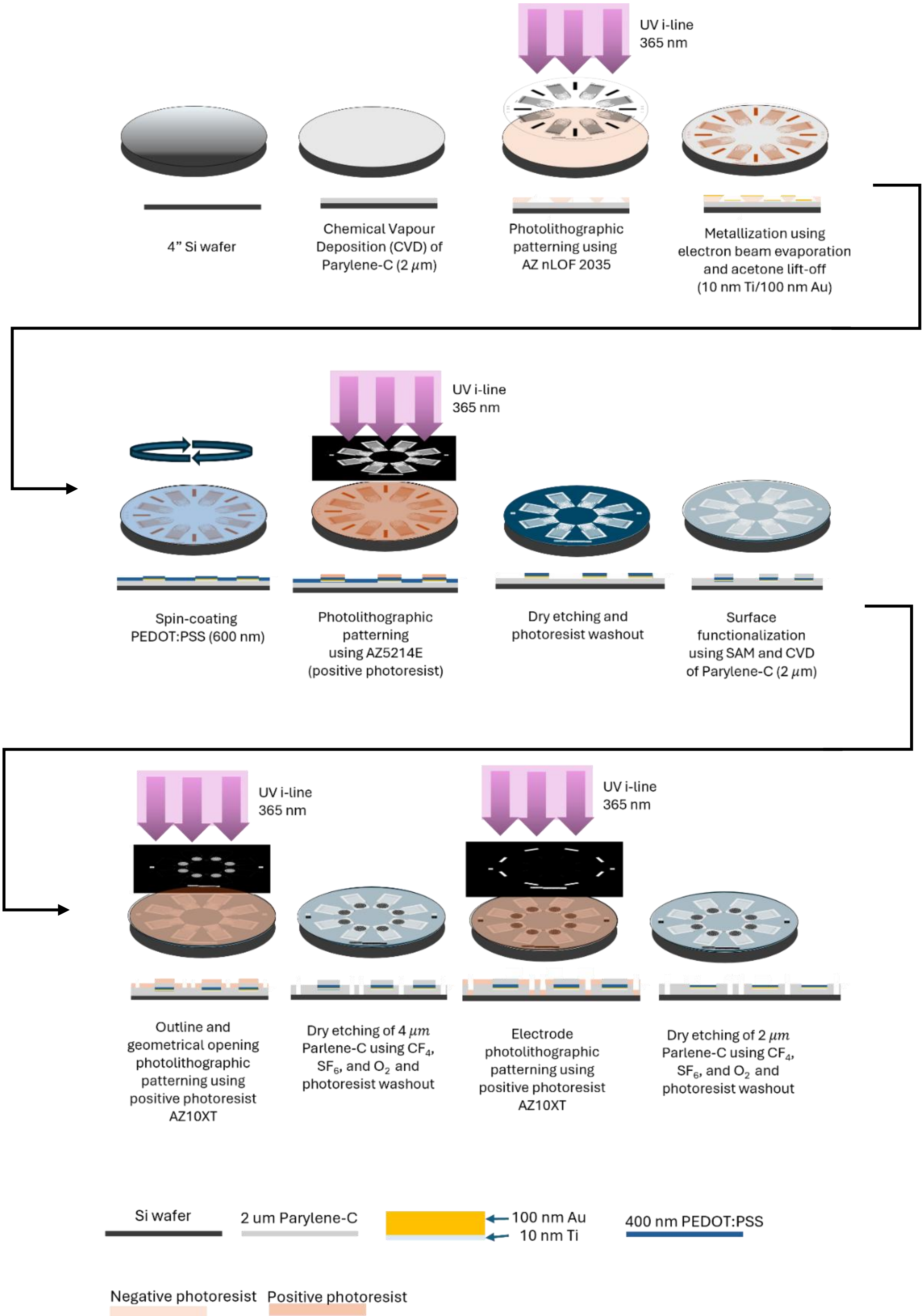

Figure S1. Microfabrication pipeline for Neuroweb devices.

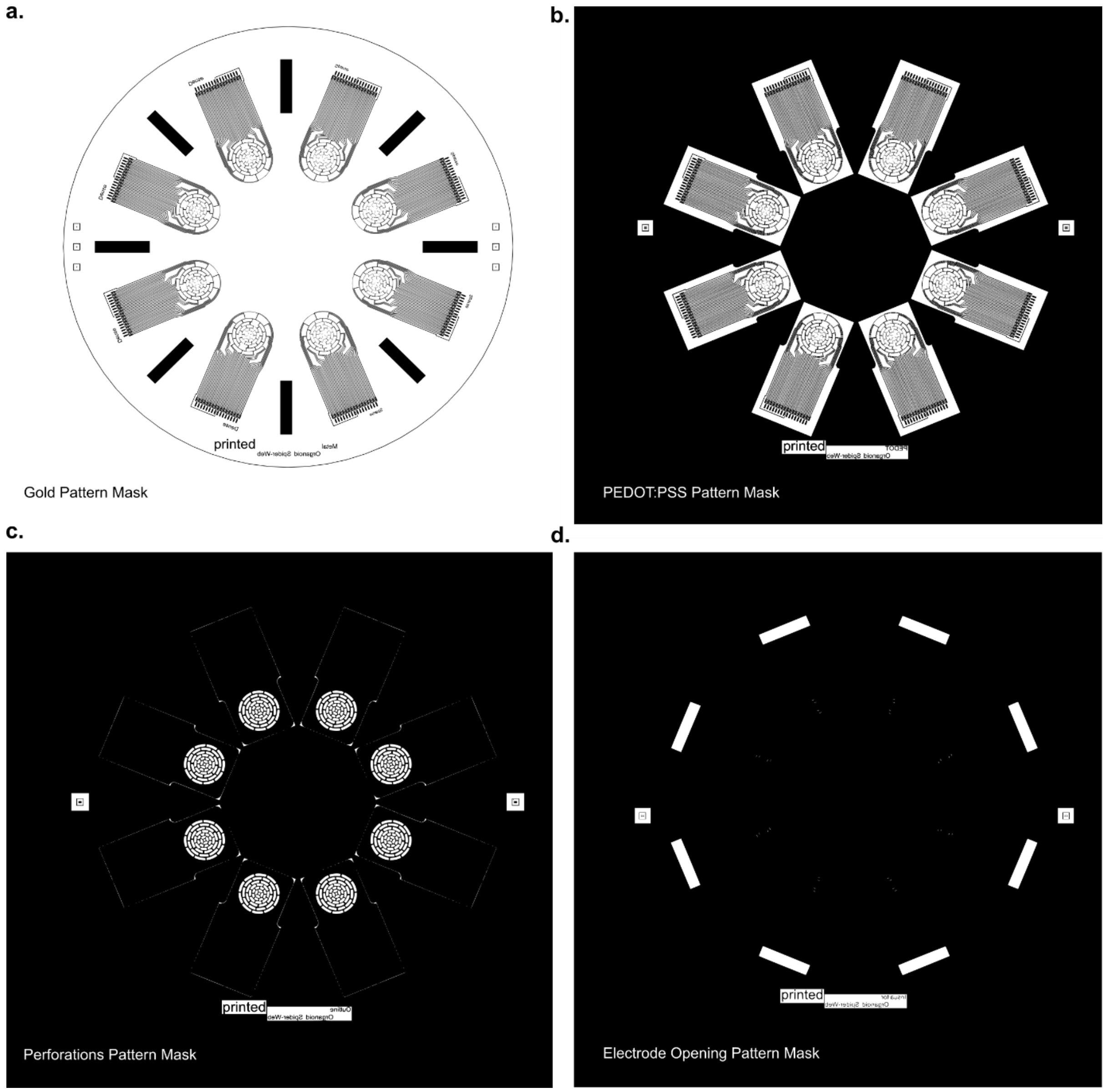

Figure S2. Positive-tone mask designs of the different layers used to fabricate Neuroweb. (a) The mask used to photolithographically pattern the areas where gold interconnects/electrodes are deposited on the silicon wafer (opaque pattern); (b) The mask used to define the areas where the PEDOT:PSS coating is subsequently etched (transparent regions); (c) The mask used to define the parylene-C regions that are etched to create a device outline that can subsequently be peeled off and the hollow geometry (transparent regions);. (d) The mask used to define the parylene-C regions that would be etched (transparent regions) to open the pads for the flat flexible cable and the electrodes at the centre (inset).

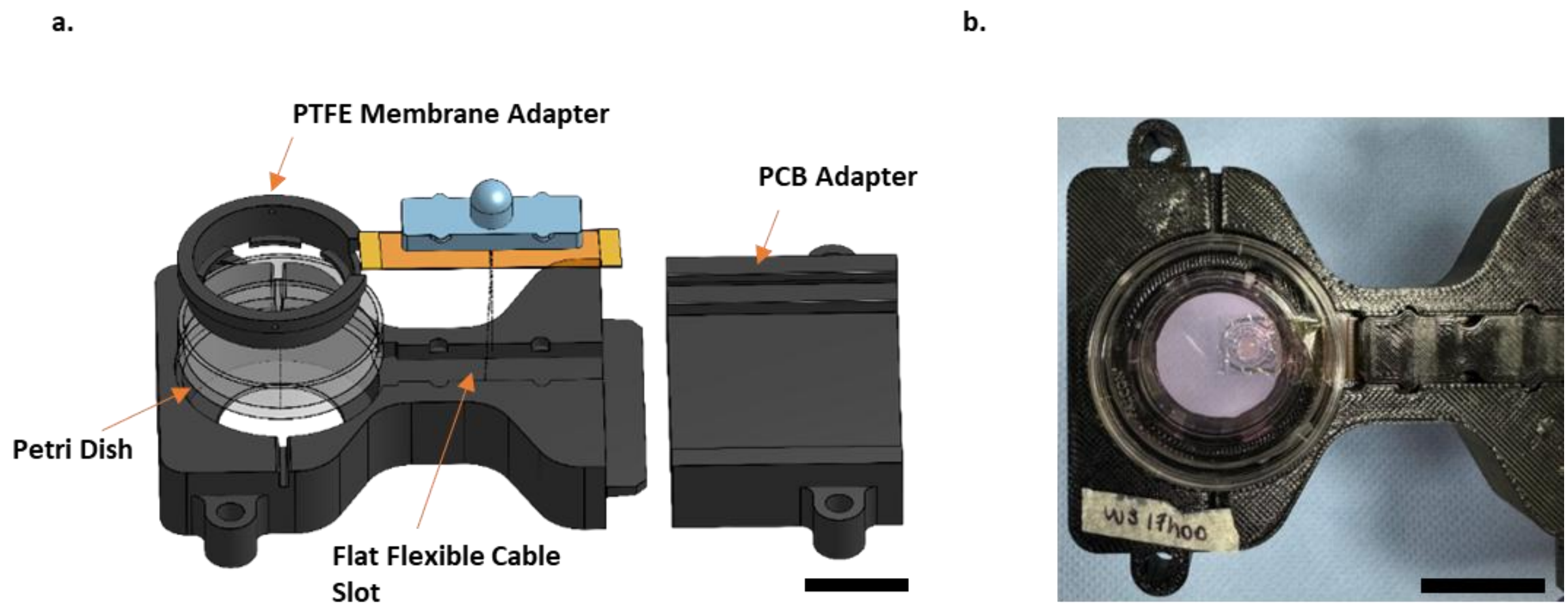

Figure S3. Holder design and setup for data acquisition. (a) Zoomed-in view of the holder used to setup the device at the air-liquid interface; (b) Optical images showing the physical setup with the device and organoid setup for long-term measurements. Scale bars: (a,b) 20 mm.

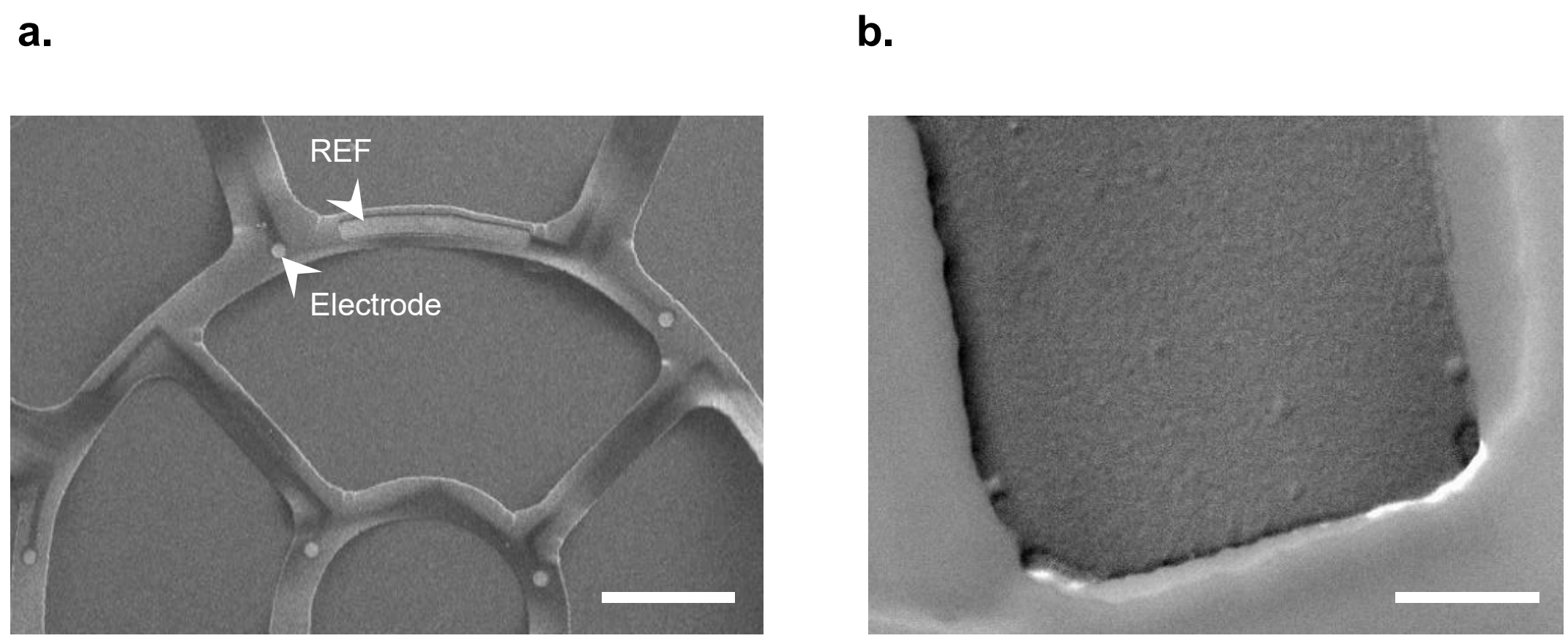

Figure S4. Scanning electron microscope (SEM) image of Neuroweb. (a) 100x magnification shows the geometry and validates the opening of the recording electrodes and reference (REF) opening of Neuroweb (brighter regions) and insulation of the interconnect; (b) 5000x magnification at the reference electrode shows a textured morphology of PEDOT:PSS. Scale bars: (a) 300 µm and (b) 10 µm.

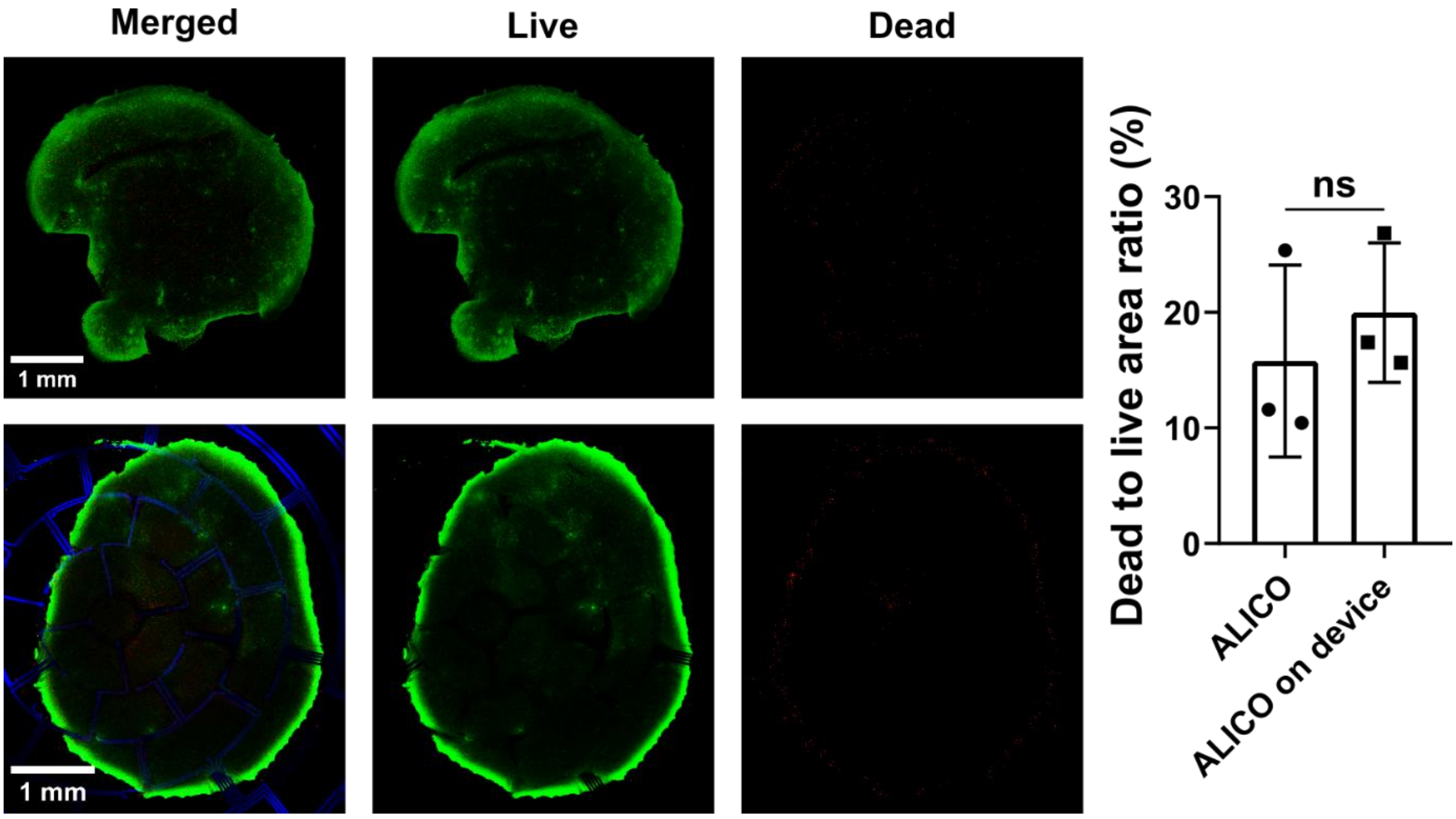

Figure S5. Live/dead staining of ALI-Cos. Example images of an ALI-CO cultured on a PTFE cell culture insert compared to an ALI-CO cultured on a Neuroweb. Green indicates live cells (Calcein-AM), while red indicates dead cells (Ethidium homodimer-1). The Neuroweb is visible in blue. n = 3 independent repeats. Two-sided t-test. ns = not significant. Scale bars: 800 µm.

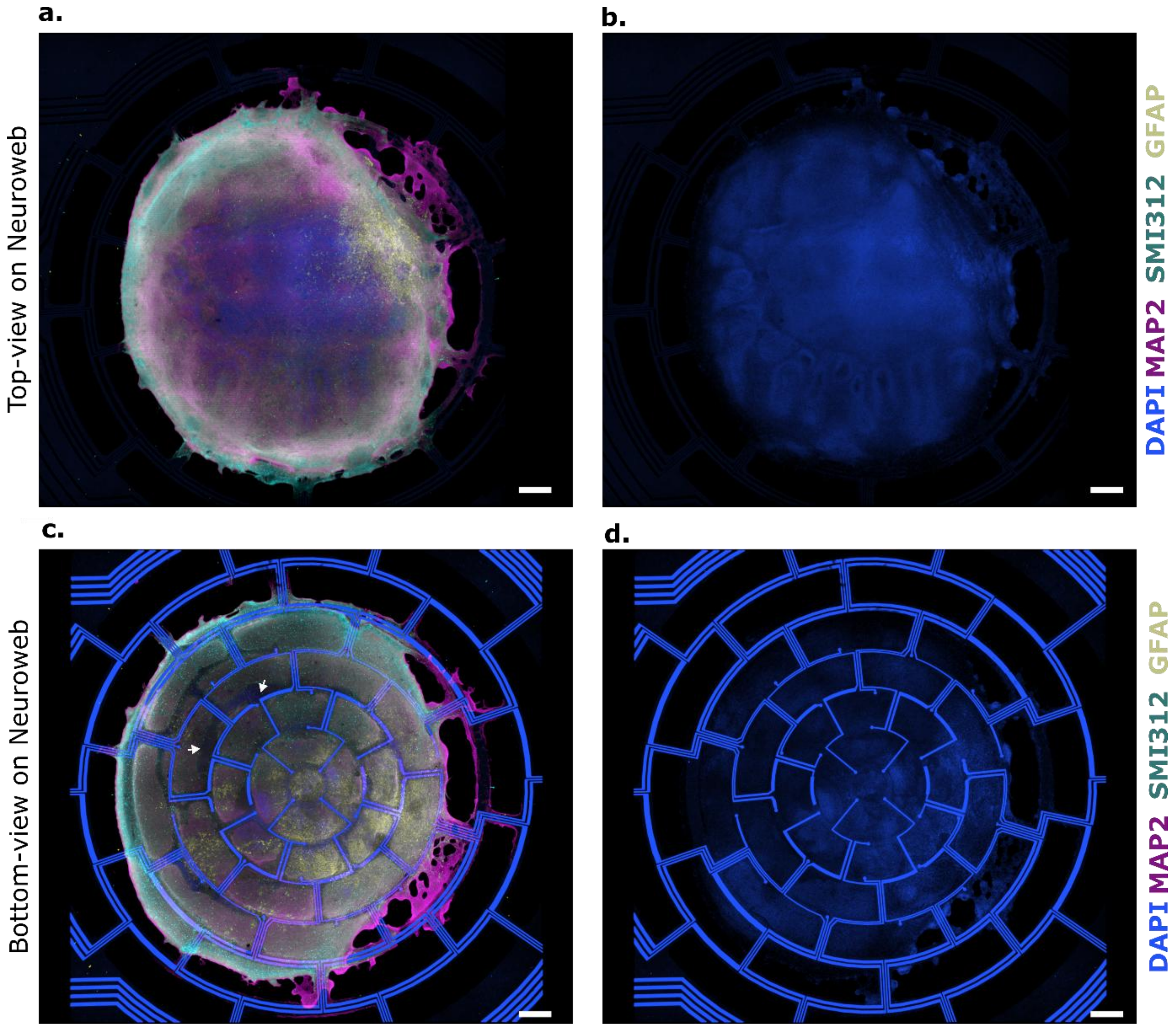

Figure S6. Immunofluorescence images of top (a-b) and bottom (c-d) sides of the ALI-CO mounted on Neuroweb (DIV 110). Arrows highlight imaging artefacts due to sample movement during staining (c). Scale bars: 500 µm.

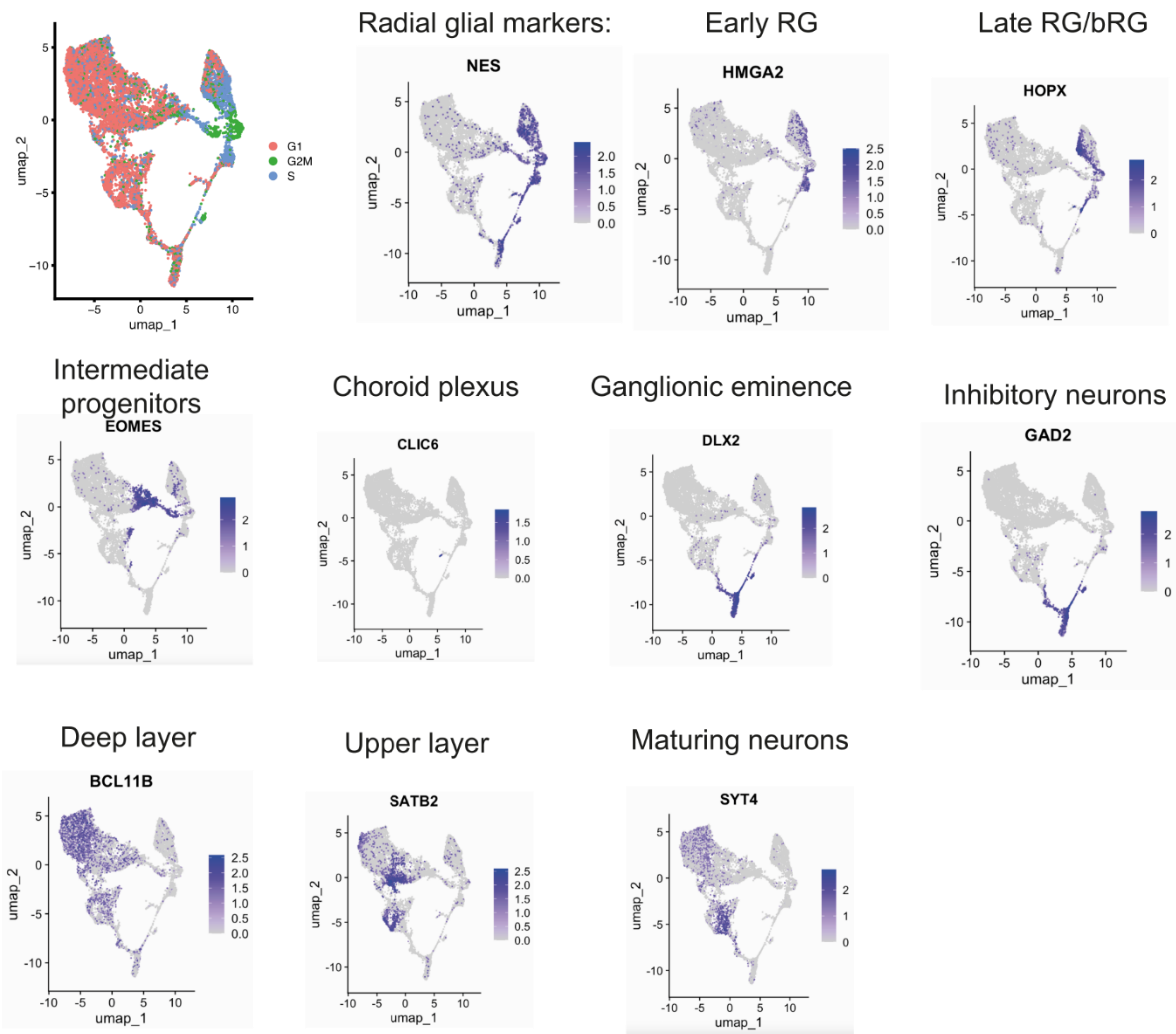

Figure S7. U-MAP showing the distribution of various proliferative states in ALI-COs.

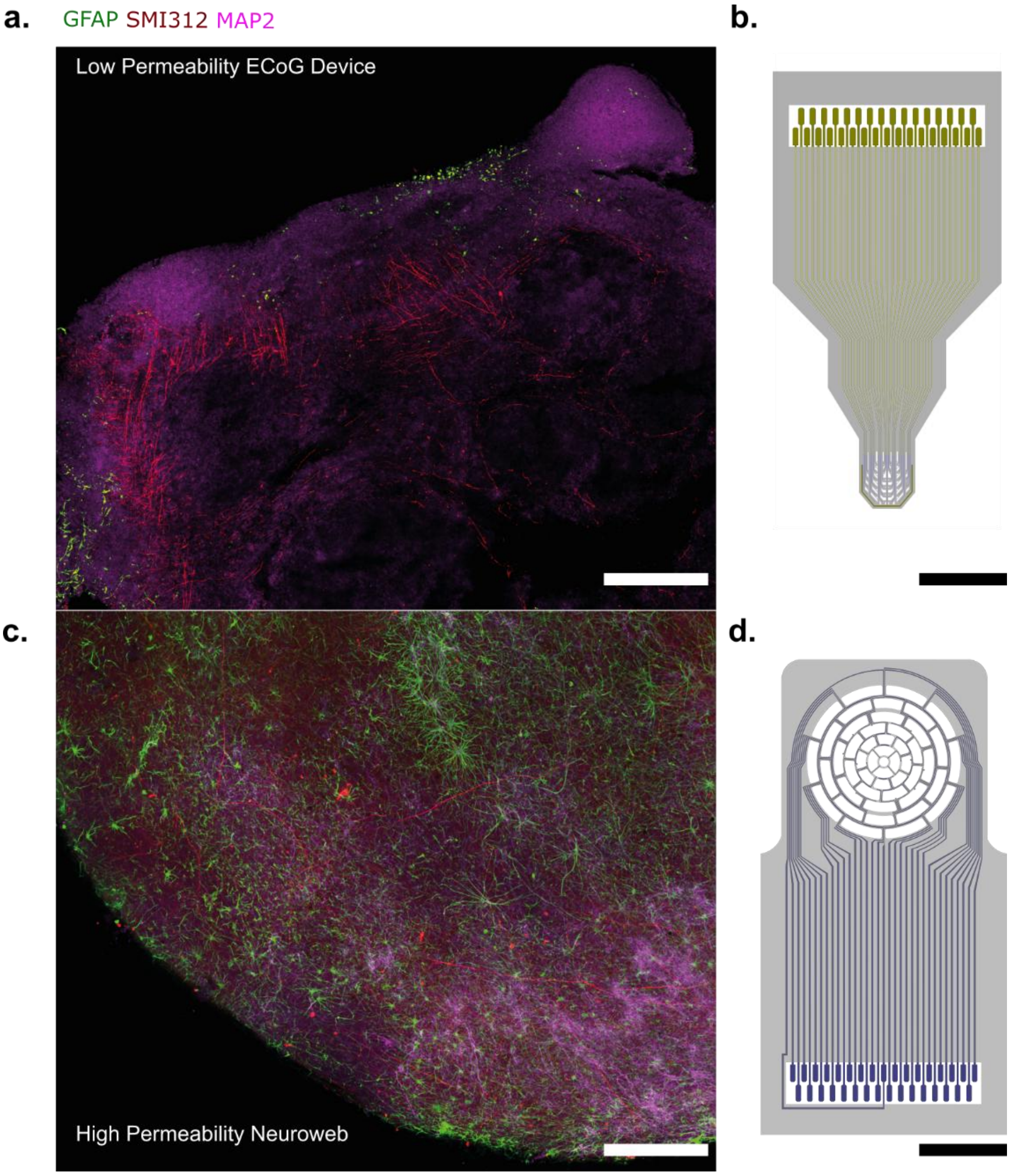

Figure S8. Immunofluorescent staining of ALI-COs on µECoG devices designed with 70% fill ratio (a-b) versus Neuroweb's 30% fill ratio (c-d). Scale bars: (a,c) 200 µm and (b,d) 5 mm.

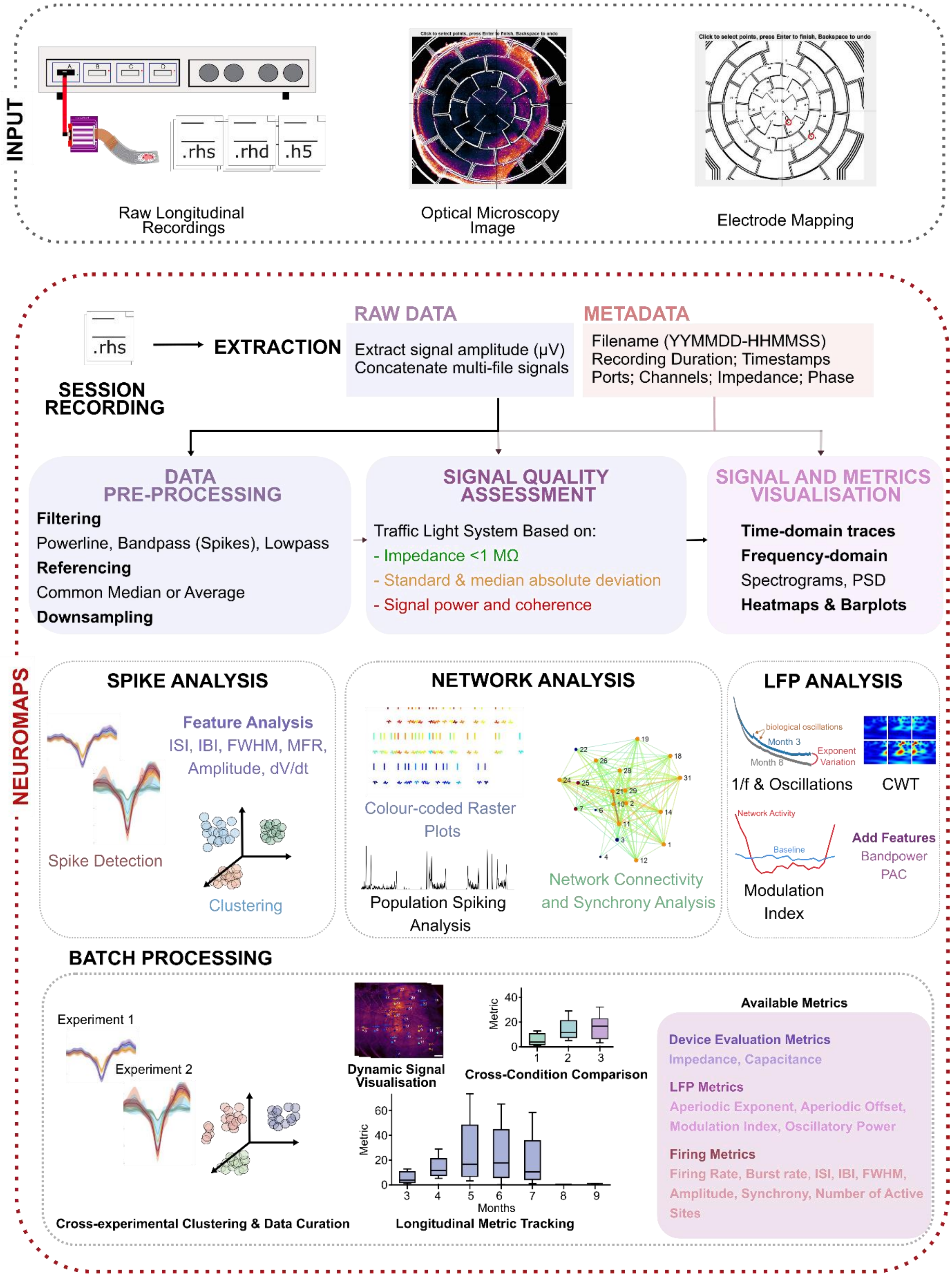

Figure S9. NeuroMaps initialisation and processing pipeline

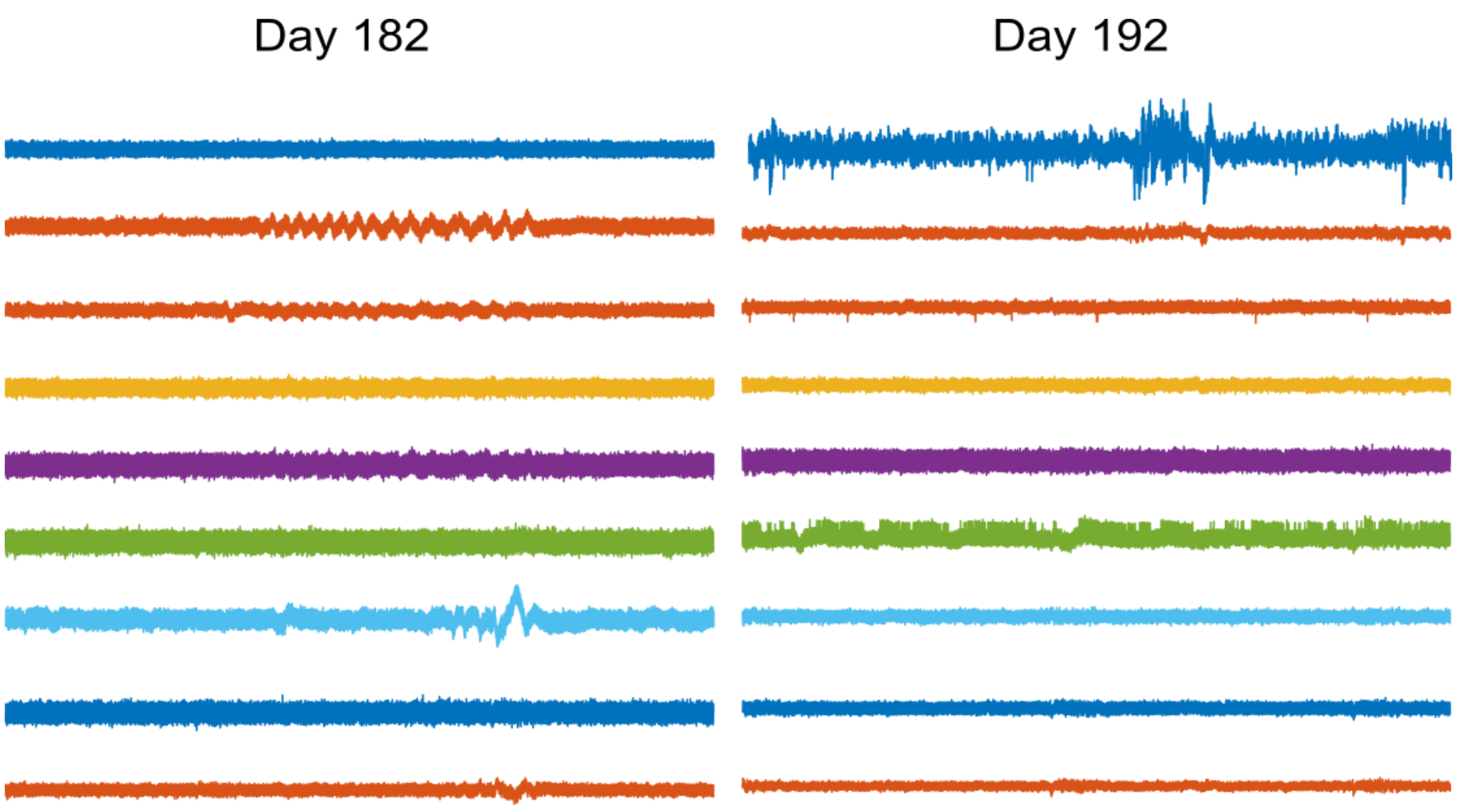

Figure S10. Signal traces detected from ALI-COs on DIV 182 and DIV 192.

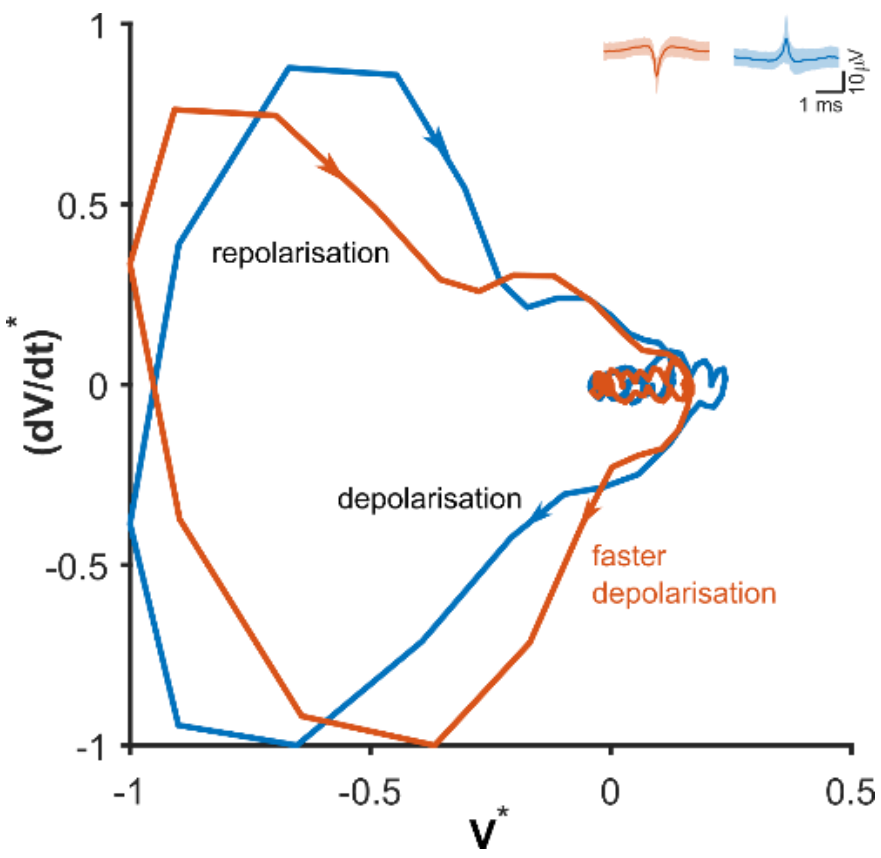

Figure S11. Normalised phase-plot showing the depolarisation speeds of positive versus negative polarity action potentials.

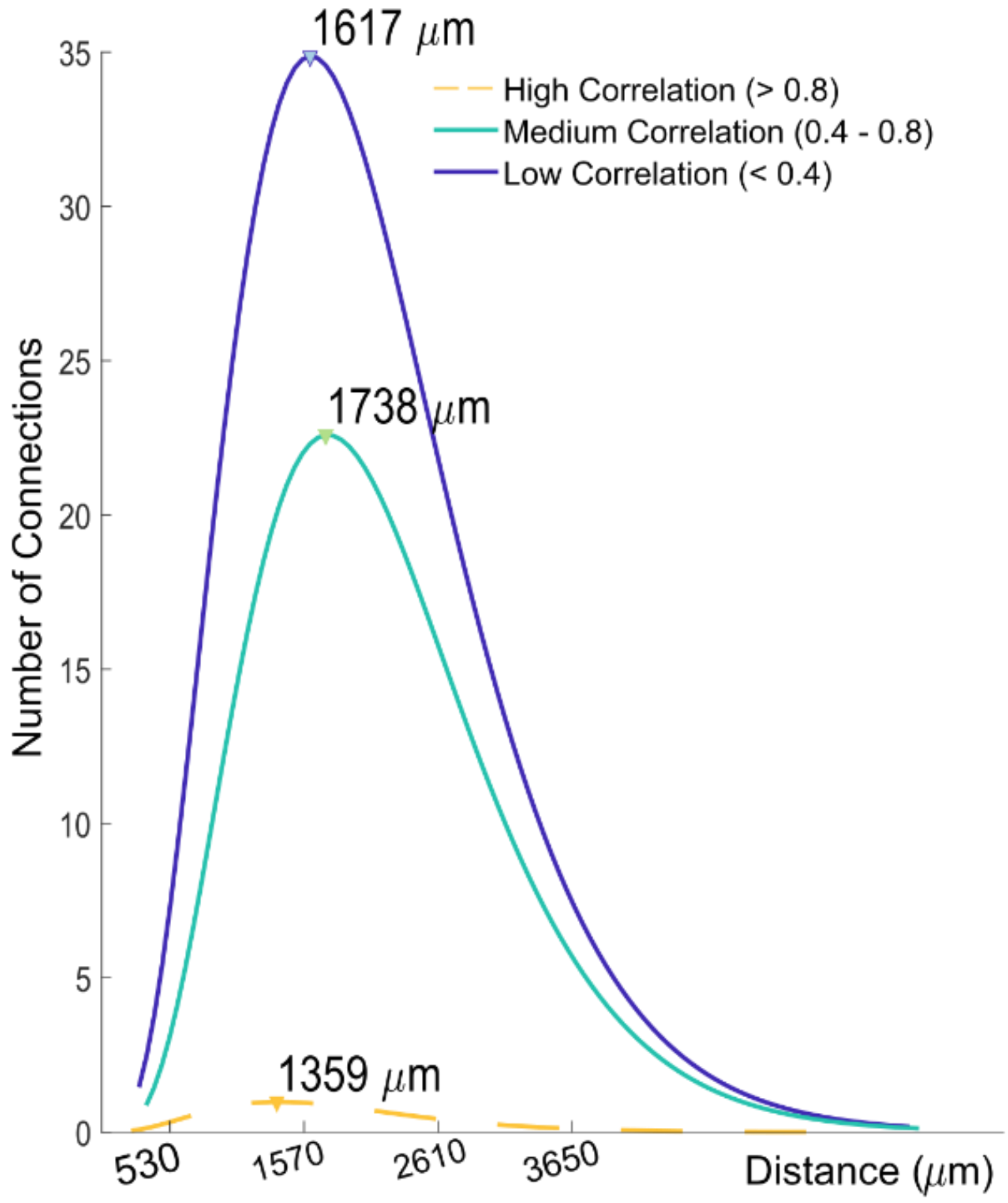

Figure. S12. Distribution of the distance between the functionally connected electrodes depending on whether the network connections exhibit low, medium, or high correlation based on their spike times. The peak of the highly correlated electrodes was at 1359 µm, the peak of moderately correlated activity occurs at 1617 µm, and the peak of weakly correlated electrodes occurred at 1738 µm.

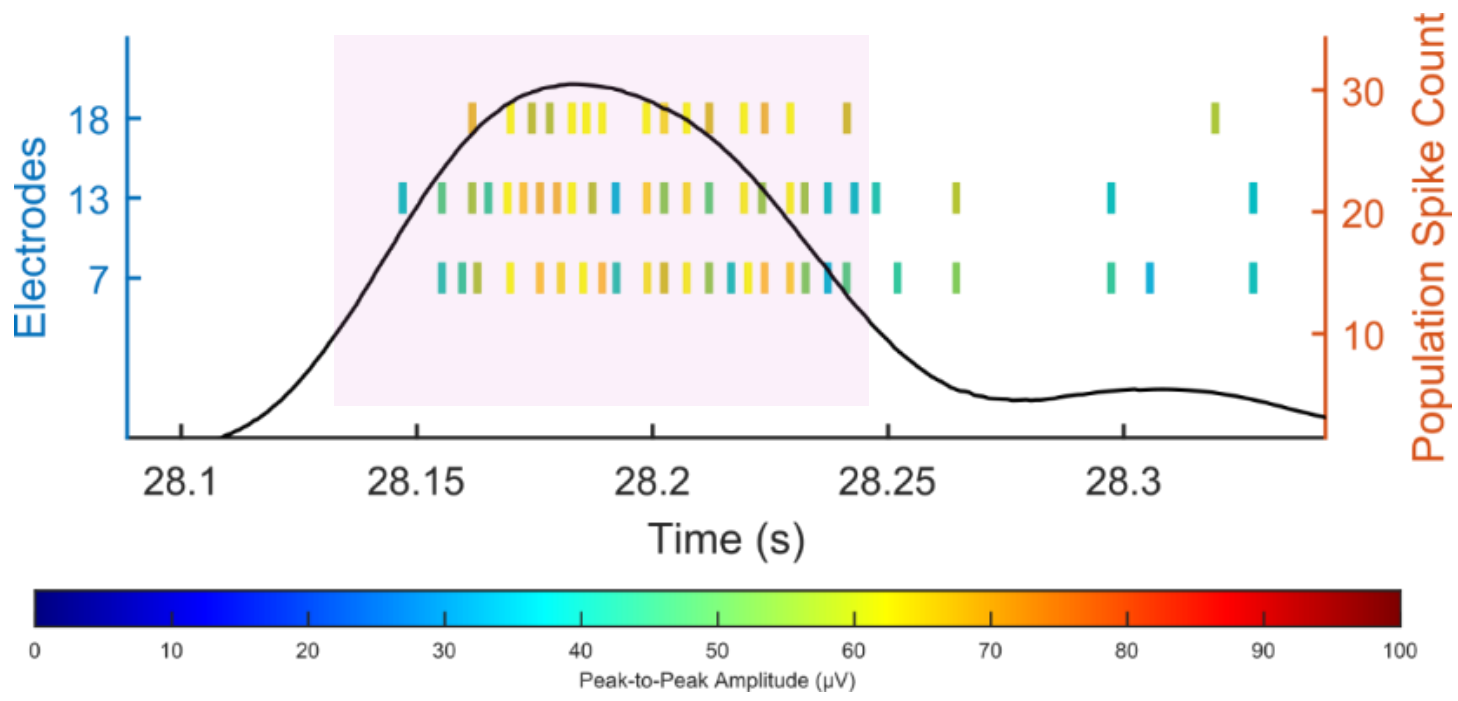

Figure S13. Zoomed-in network population spiking plot overlayed on raster plot showing the multiple spikes that contribute to the population spike count within a 0.1-second Gaussian window.

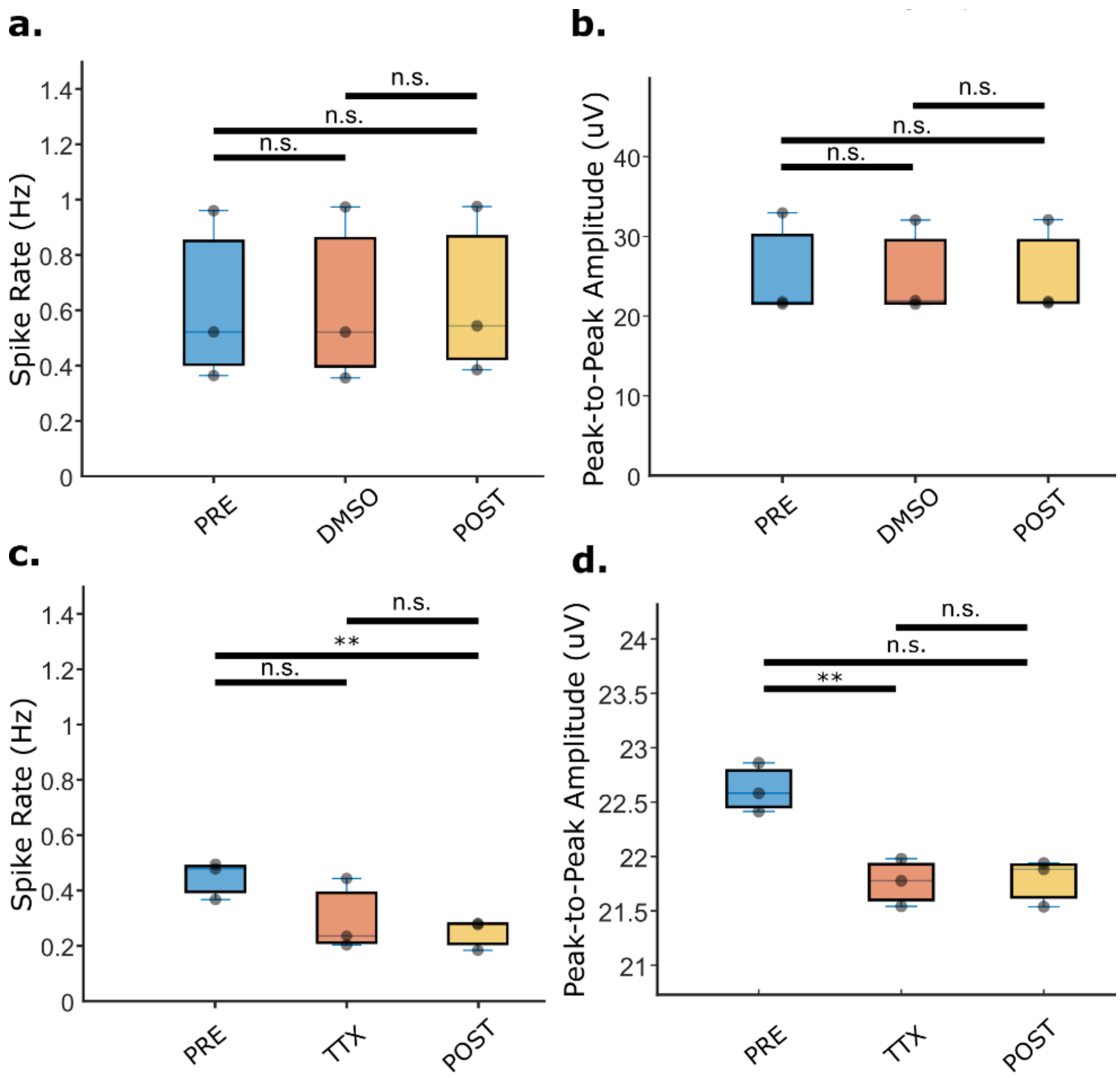

Figure S14. Pharmacological modulation of spontaneous electrophysiological activity in brain organoids on planar MEAs. Spike rate and peak-to-peak spike amplitude were quantified across baseline (PRE), acute pharmacological treatment, and post-treatment phases. (a,b) Vehicle control with DMSO produced no significant change in spike rate or spike amplitude relative to baseline. (c,d) Tetrodotoxin (TTX) significantly reduced spike rate and peak-to-peak amplitude compared to baseline (PRE), consistent with a partial suppression of voltage-gated sodium channel-dependent activity. N = 3, with each point represents one ALI-CO (DIV 140). Statistical comparisons were performed using repeated measures one-way ANOVA with Geisser-Greenhouse correction and Tukey multiple comparison to track the same organoids across recording conditions. **$p<0.01$, n.s. = not significant. Spikes with amplitudes below 10 μV were excluded from the analysis.

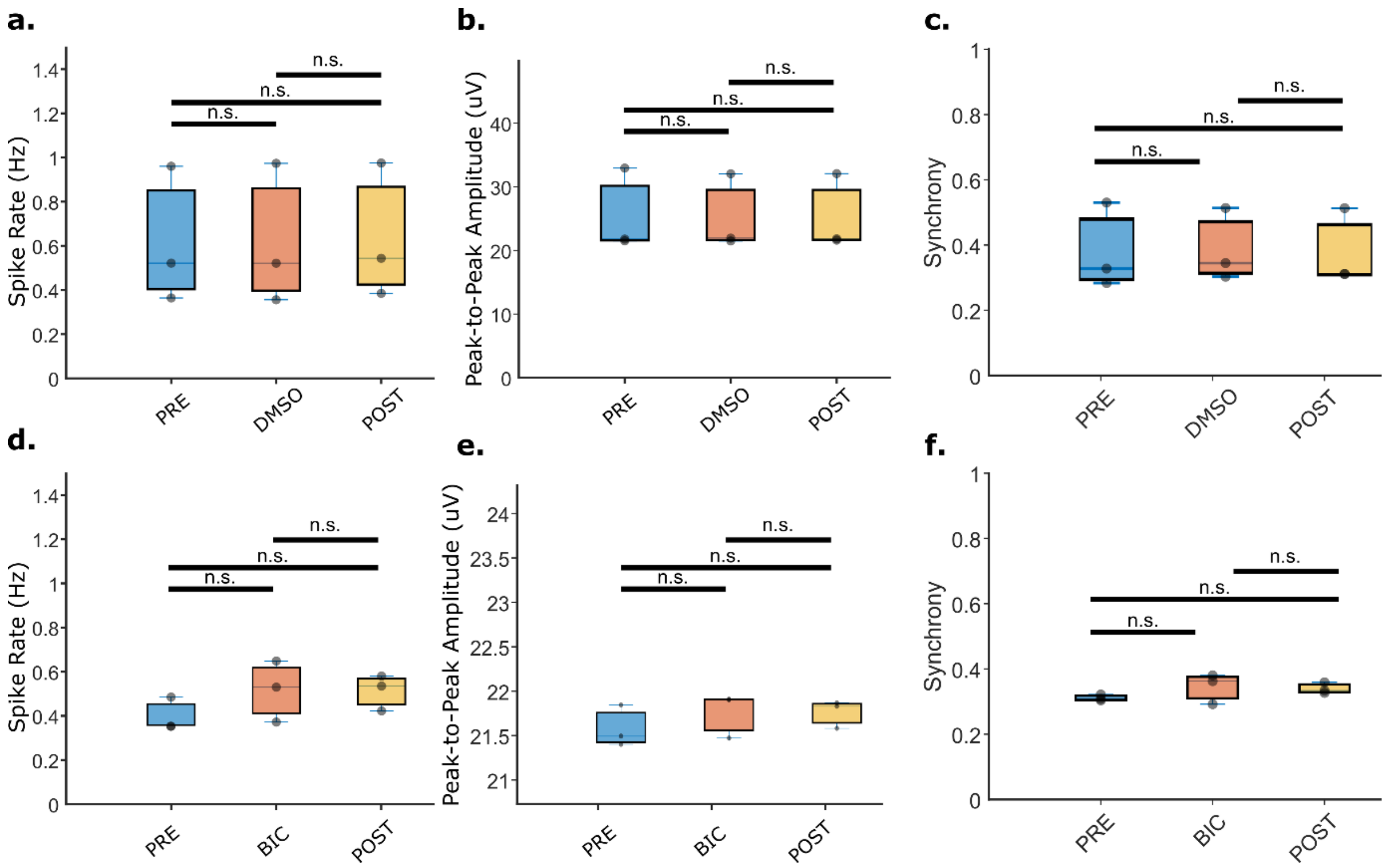

Figure S15. Pharmacological modulation exploring GABAergic maturation in brain organoids (DIV 140) on planar MEAs. Spike rate, peak-to-peak spike amplitude, and synchrony were quantified across baseline (PRE), acute pharmacological treatment, and post-treatment phases. (a,b,c) Vehicle control with DMSO produced no significant change in spike rate or spike amplitude relative to baseline. (d,e,f) Bicuculline (BIC) showed a modest yet not statistically significant increase in all metrics following treatment. N = 3, with each point represents one ALI-CO (DIV 140). Statistical comparisons were performed using repeated measures one-way ANOVA with Geisser-Greenhouse correction and Tukey multiple comparison to track the same organoids across recording conditions. n.s. = not significant. Spikes with amplitudes below 10 µV were excluded from the analysis.

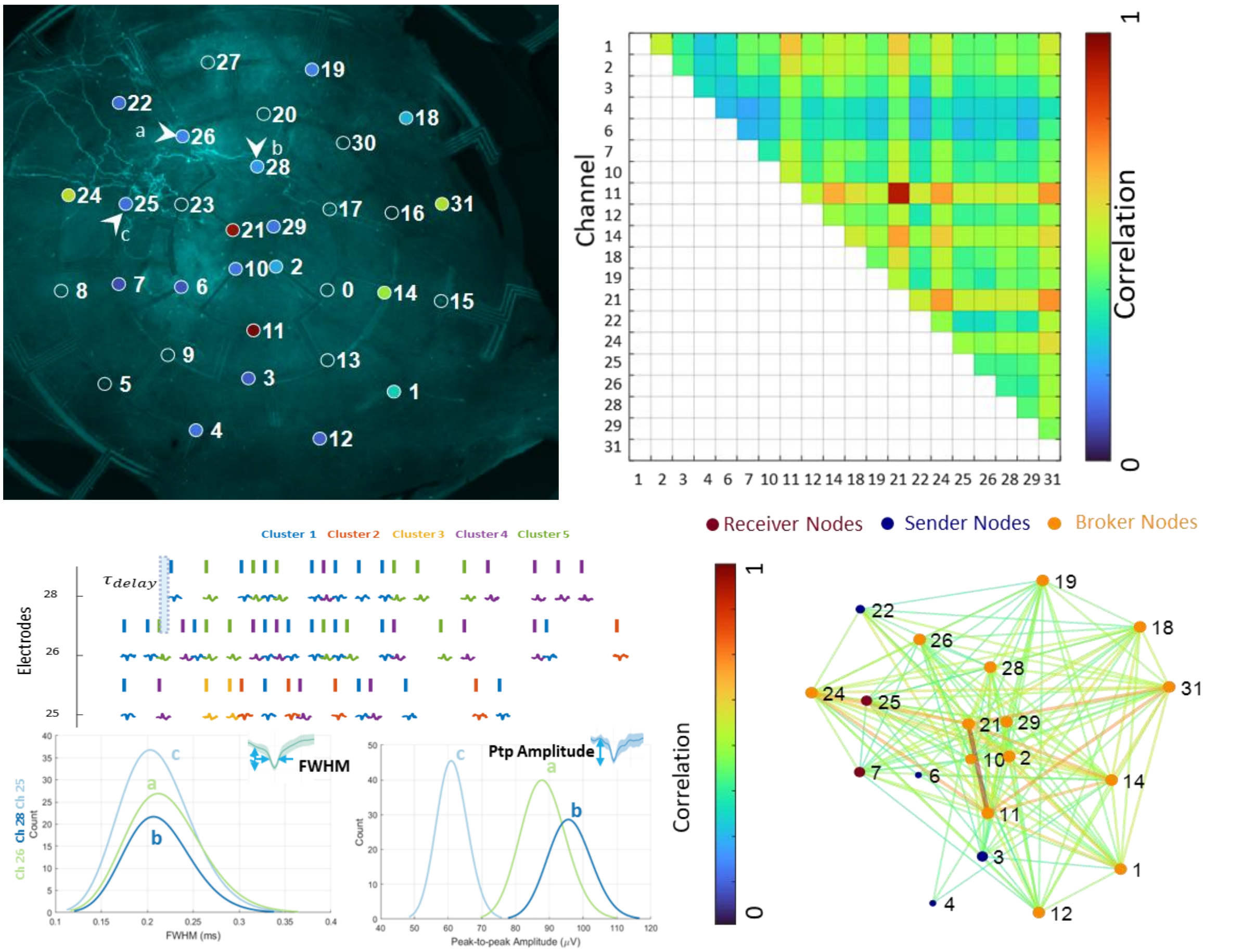

Figure S16. Sample localised spike feature and network analysis at DIV 110. (a) Localised visualisation of the spiking activity. (b) Correlation heatmap between the multiple channels shown in (a). (c) Spike time-tiling correlation (STTC) analysis of the organoid mapped in (a) reveals a medium-to-high correlation between most electrodes. Clustering of detected spikes and feature analysis shows that the spikes from electrodes 25, 26, and 28 exhibit a similar FWHM distribution with varying peak-to-peak amplitudes. The highest amplitude was detected at electrode 28 followed by electrodes 26 and 25. This is consistent with the network analysis, which shows that electrode 25 is a receiver electrode while electrode 28 and 26 are broker electrodes, suggesting the flow of spikes through the axon from electrode 28 to 26 and finally 28.

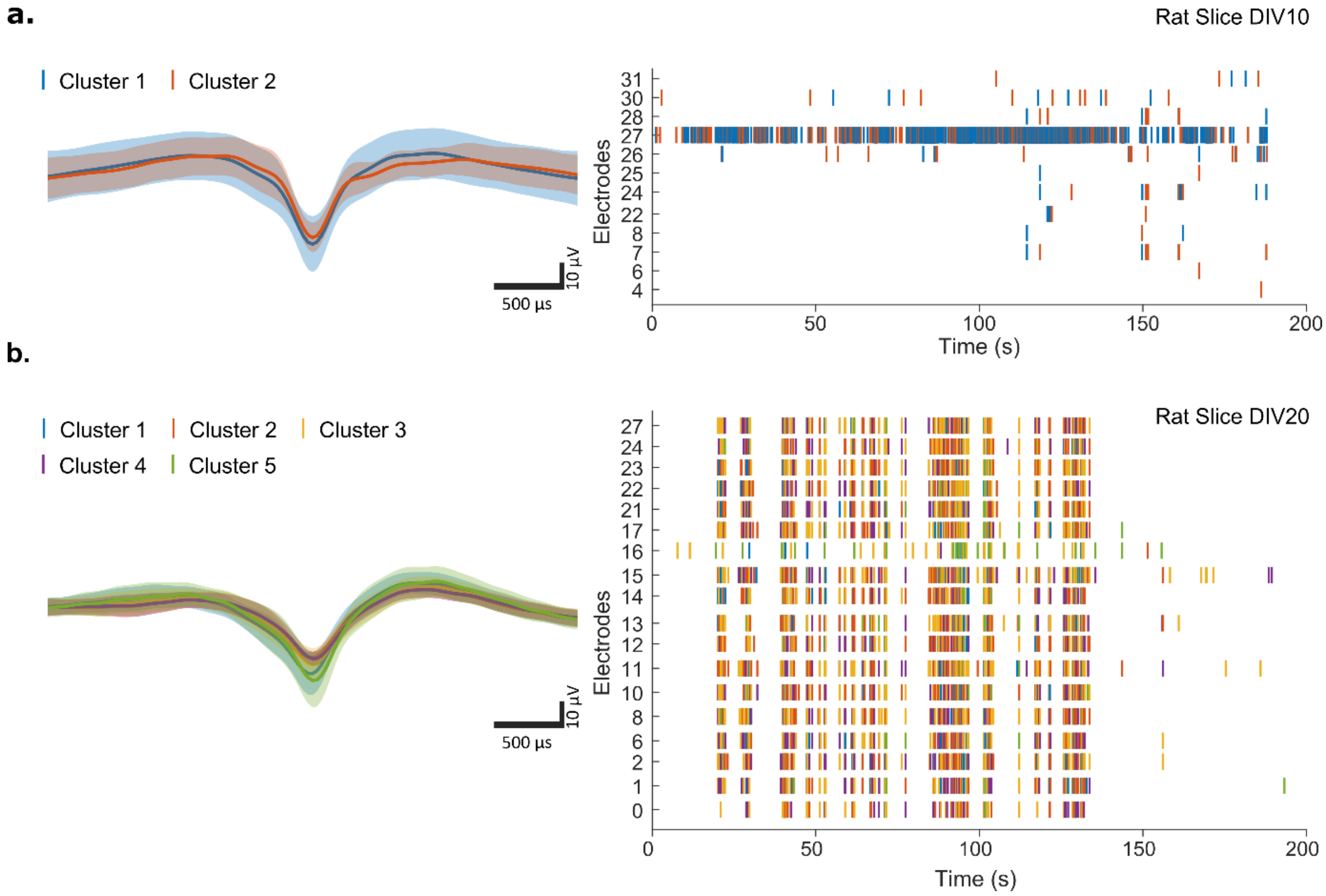

Figure S17. Action potentials and raster plots obtained from acute rat slice measurements. (a) Sparse activity was detected from P5 rat slices at DIV 10, with two clusters detected. (b) Structured activity and frequent bursts were detected from P5 at DIV 20, with 5 waveform clusters detected. Waveform and raster colours depict the corresponding clusters.

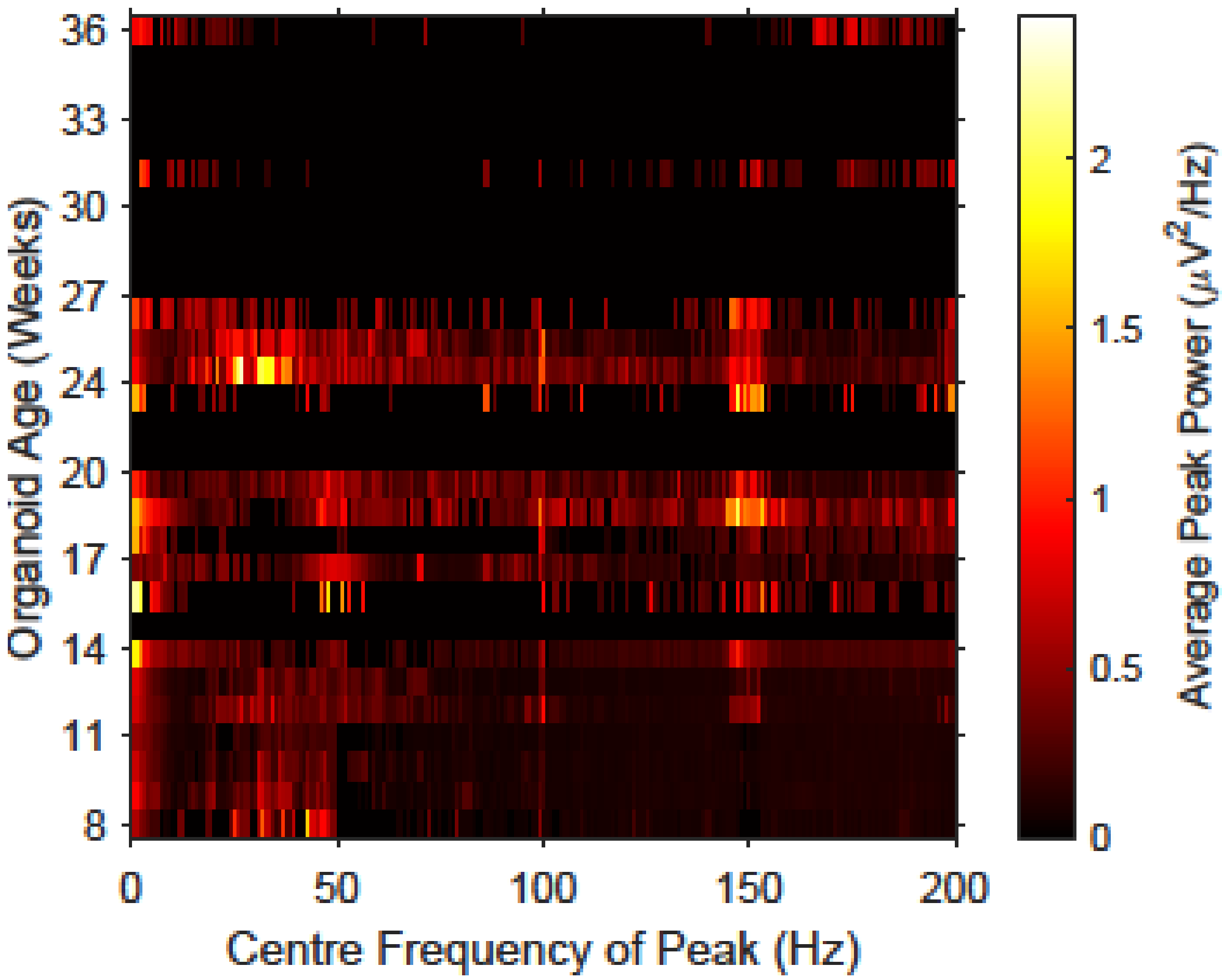

Figure S18. Heatmap illustrating the average peak power of various oscillatory centre frequencies obtained from FOOOF analysis across ALI-CO maturation, demonstrating an increase in broadband gamma activity.

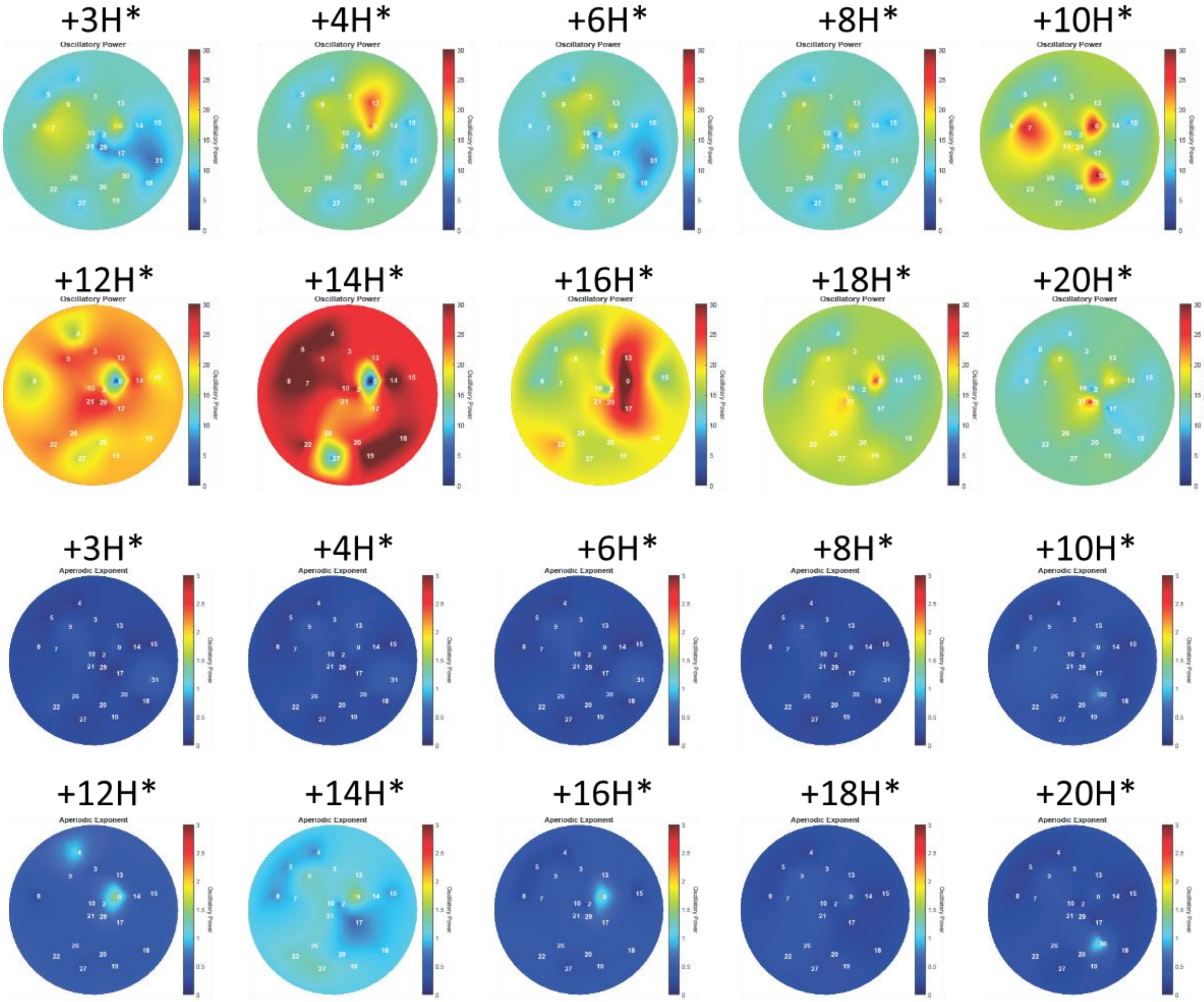

Figure S19. Topographic maps illustrating changes in the oscillatory power of the various electrodes and the aperiodic exponent as a function of media changes, revealing potential effects of metabolic activity.
